# Marine biomaterials in biomedical nano/micro-systems

**DOI:** 10.1186/s12951-023-02112-w

**Published:** 2023-11-05

**Authors:** Yanan Wang, Long Chen, Yuanzheng Wang, Xinyuan Wang, Deyao Qian, Jiahui Yan, Zeyu Sun, Pengfei Cui, Liangmin Yu, Jun Wu, Zhiyu He

**Affiliations:** 1 Frontiers Science Center for Deep Ocean Multispheres and Earth Systems, Key Laboratory of Marine Chemistry Theory and Technology, Ministry of Education/Sanya Oceanographic Institution, Ocean University of China, Qingdao, 266100 China; 2 Frontiers Science Center for Deep Ocean Multispheres and Earth Systems, Key Laboratory of Marine Chemistry Theory and Technology, Ministry of Education/Sanya Oceanographic Institution, Ocean University of China, Sanya, 572024 China; 3 Department of Orthopedics, Guizhou Provincial People’s Hospital, Guiyang, 55000 Guizhou China; 4 College of Marine Life Sciences, Ocean University of China, Qingdao, 266100 China; 5 Division of Life Science, The Hong Kong University of Science and Technology, Hong Kong SAR, 999077 China

**Keywords:** Chitosan-based nanoparticles, Alginate hydrogels, Collagen-based nanocomposites, marine fatty acid-based nanomaterials, Marine biomineralized nanocomposites

## Abstract

Marine resources in unique marine environments provide abundant, cost-effective natural biomaterials with distinct structures, compositions, and biological activities compared to terrestrial species. These marine-derived raw materials, including polysaccharides, natural protein components, fatty acids, and marine minerals, etc., have shown great potential in preparing, stabilizing, or modifying multifunctional nano-/micro-systems and are widely applied in drug delivery, theragnostic, tissue engineering, etc. This review provides a comprehensive summary of the most current marine biomaterial-based nano-/micro-systems developed over the past three years, primarily focusing on therapeutic delivery studies and highlighting their potential to cure a variety of diseases. Specifically, we first provided a detailed introduction to the physicochemical characteristics and biological activities of natural marine biocomponents in their raw state. Furthermore, the assembly processes, potential functionalities of each building block, and a thorough evaluation of the pharmacokinetics and pharmacodynamics of advanced marine biomaterial-based systems and their effects on molecular pathophysiological processes were fully elucidated. Finally, a list of unresolved issues and pivotal challenges of marine-derived biomaterials applications, such as standardized distinction of raw materials, long-term biosafety in vivo, the feasibility of scale-up, etc., was presented. This review is expected to serve as a roadmap for fundamental research and facilitate the rational design of marine biomaterials for diverse emerging applications.

**Graphical Abstract:**

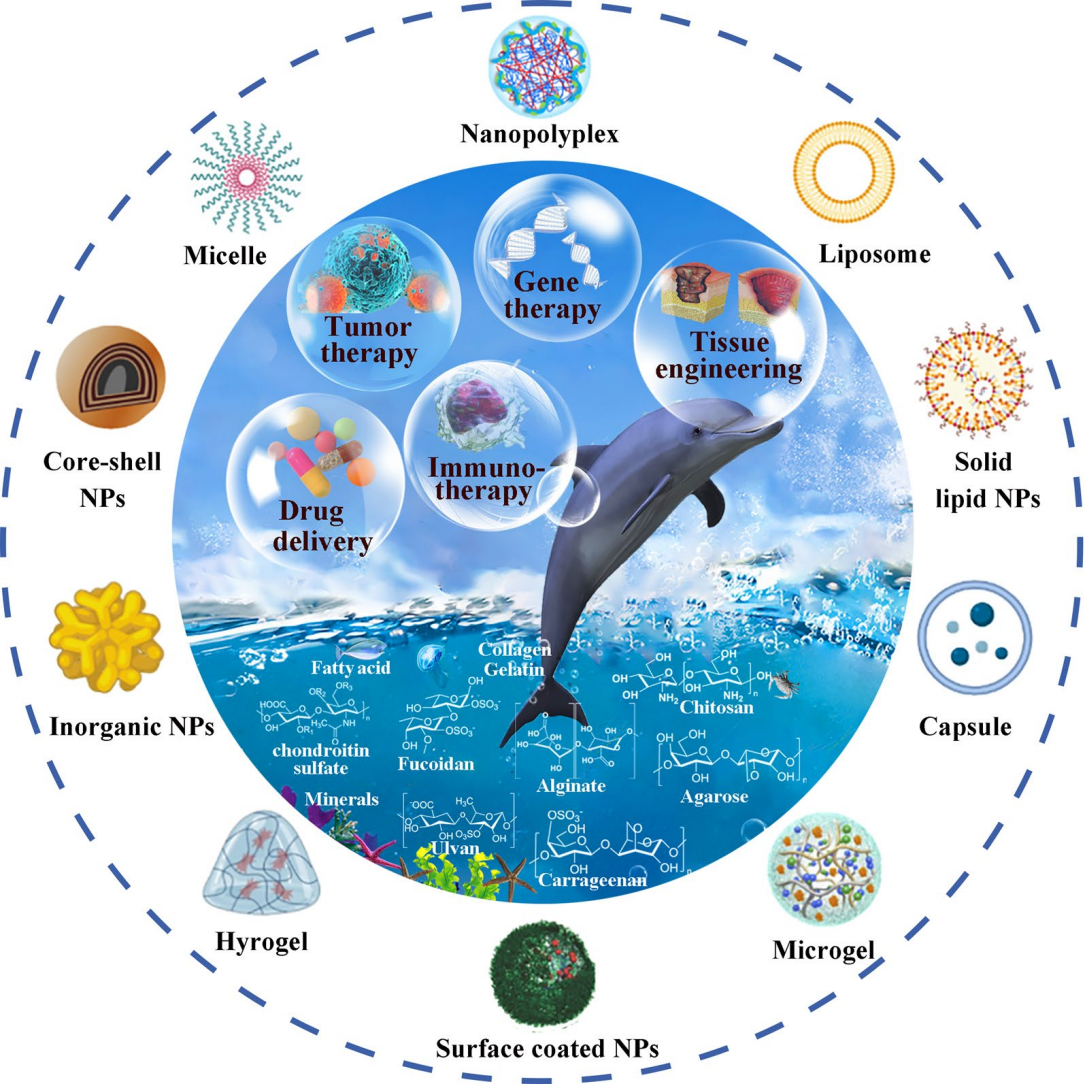

## Introduction

The ocean ecosystem, which is the largest on Earth, is an abundant source of materials [1]. Marine biomaterials research is experiencing a renaissance as the technological revolution has significantly improved the efficiency with which these renewable natural resources are utilized. Numerous studies demonstrate that the construction and applications of biomaterials derived from marine organisms have been rapidly explored since their first use as anti-tumor agents in 1967 (i.e., holothurin, the earliest biologically active substance extracted from marine origin) [2]. Marine resources (fish by-products, shell sources, and marine macroalgae) provide large-scale, cost-effective supplies of natural materials used in a variety of biomedical applications [3]. Particularly, the re-separated biomaterials from fish waste may be beneficial for avoiding environmental issues and manufacturing products with added value, thereby facilitating the economic return of marine processing industries.

Researchers have invested efforts in the design and fabrication of innovative engineering nanomaterials with improved properties that could be applied in a variety of applications, including pharmaceutical formulations, controlled drug delivery, therapeutic and diagnostic aids, and tissue engineering scaffolds, among others [4, 5]. Notable characteristics of materials derived from marine natural resources include biocompatibility, ease of modification, low-immunogenicity, gel-forming properties, and abundance of sources, all of which are important in biomedicine applications [6]. Moreover, due to the high salinity, low temperature, minimal temperature difference, and limited dissolved oxygen and light of the seawater buffer system, marine organism-derived polysaccharides evidently differ in structure, content, and biological activities from polysaccharides derived by terrestrial species, especially their diverse biomedical therapeutic effects, which include antitumor, antibacterial, antioxidant, anti-inflammatory, immunomodulatory, and cardioprotective effects, as well as promoting cellular migration, cell–matrix interactions, and tissue regeneration, etc. [7–10]. On the basis of these unique general advantages, those marine components have expanded applications in the biomedical and pharmaceutical fields in recent years, especially in the advanced field of drug delivery.

In marine ecosystems, over 25,000 biologically active compounds have been identified. Indeed, prior research indicates that marine materials purified from marine species fall into the categories of polysaccharides (such as chitosan, alginate, chondroitin sulfate (CS), carrageenan, fucoidan, and agarose), natural protein components (collagen, gelatin, etc.), marine secondary metabolites, and marine minerals (hydroxyapatite). Due to their diverse biofunctions, marine-derived polysaccharides are the most prevalent and naturally available biopolymers and have garnered more popularity than synthetic polymers [11, 12]. Several polysaccharides (e.g., chitosan) are widely recognized as excellent bio-adhesive matrices that are conducive to enhancing the bio-adhesion towards mucous membranes and epithelia, resulting in superior transmucosal transport and absorption [13, 14]. Due to their gelling properties, polysaccharides such as alginate, carrageenan, ulvan, agarose, etc., can be utilized to generate ionotropic or thermotropic gels for prolonged drug delivery or wound dressing, etc. [15–18]. Besides, CS and fucoidan have been identified as targeting moieties that facilitate the precise delivery of drugs to lesions [19–21]. Due to their numerous modifiable groups, such as hydroxyl, carboxyl, and amino groups, etc., these natural polymers are particularly receptive to chemical or biochemical modification to produce functional derivatives. These derived groups may play essential roles in the formation of covalent or non-covalent bonds with the cell surface, tissues, and specific organs. For nanostructure-based therapies, such as targeted delivery, sequential targeting, stimuli-response, etc., several functional conjugates or stimuli-sensitive chemical bonds could also be introduced into these polysaccharides [22]. Combining the unique characteristics (highly stable, safe, biocompatible, biodegradable, nontoxic, low cost, and abundant) of the majority of marine polysaccharides with the specific bioactivities (site-specific targeting, receptor binding, mucosal adhesion and penetration, etc.) of several polysaccharides, marine polysaccharide-based nanomaterials are an excellent source for nanotechnology applications. Natural protein components, such as collagen or gelatin extracted from fish byproducts (fish skin, fish scales, or other fishery wastes) was combined with other biomaterials to fabricate 3D hydrogel networks that provide structural support for cells and maintain the homeostasis of their microenvironment during morphogenesis and differentiation [23, 24]. The lipophilic compounds (polyunsaturated fatty acids (PUFAs)) from marine fish oil have been employed to stabilize nanodroplets for the construction of a liposome or nano-emulsifying system, where the nutritional and health benefits of PUFAs are undisputed [25, 26]. In addition, marine biominerals (such as calcium carbonates and calcium phosphate) have greater potential for use as bone-remodeling formulations in orthopedics and dentistry due to their mechanical stability, porosity and ability to stimulate tissue regeneration [27]. Due to their unique properties, these marine biomaterials are appropriate for the fabrication of various multifunctional nanosystems or hydrogels with diverse functions, excellent biodegradability and biocompatibility and can be employed in drug delivery and regenerative medicine.

In this review, we aim to provide a comprehensive overview of recent advancements in marine biomaterial-based nano/micro-systems in the past three years, particularly emphasizing their potential in treating a range of disease. This review aims to provide a comprehensive and broad overview of marine biological materials rather than focusing on a specific single category of biomedical marine materials, with a particular emphasis on different classifications such as marine polysaccharides, proteins, minerals, and fatty acids. The structural diversity and biomedical properties of these materials derived from marine organisms have led to the development of drug delivery systems for a variety of applications, including the treatment of bacterial infections, tumors, acute organ injuries, and chronic metabolic diseases. This review summarizes the synthesis, preparation, physicochemical properties, therapeutic effects, and molecular mechanisms of action of nano-drug delivery vehicles based on marine biomaterials. By elucidating the assembly principles and therapeutic mechanisms of these micro-nano delivery systems, it becomes feasible to fully maximize on their market potential and accelerate their clinical translation. Concretely, in the review of the application of per marine biomaterial-based nano/micro-systems, the physicochemical characteristics of raw materials (natural marine biocomponents) and their potential biological activities that could exert synergistic therapy effects were initially introduced. Then, we emphasized the assembly processes of engineered nanomaterials and the fundamental functions of formulation building blocks. After that, we gave a thorough overview of the pharmacokinetics and pharmacodynamics in specific disease animal model of each marine biomaterial-based nanosystem, as well as their regulation on molecular pathophysiological processes. To demonstrate the potential of these high-performance bio-based materials, we highlighted a number of illustrative marine biomaterials-related commercial products. At the final illustration of each type of marine biomaterials, a list of unresolved issues and pivotal challenges for future research was presented. We believe that the review will provide theoretical guidance for the development of intelligent marine biomaterials and their eventual clinical transformation, thereby improving public health.

## Application of marine organisms-derived polysaccharide nanomaterials in drug delivery

Marine polysaccharides, which primarily derived from marine macroalgae, microorganisms, phytoplankton, and animals, are consist of multiple monosaccharide molecules linked by glycoside bonds [1]. Marine polysaccharides have been extensively studied as therapeutic adjuvants or dietary supplements for improving diverse therapeutic benefits. Notably, the exact composition, substituent distribution and their connection with backbone, and glycosidic linkage types of those marine organisms-derived polysaccharides differ between their derived species and varies between seasons. Furthermore, the difference in physiological activities of polysaccharides from different organism is quite big. For instance, a naturally basic and positively charged material known as chitosan, which is extracted from the shells of crustaceans like crabs and shrimp, possesses exceptional biological characteristics, including antibacterial, antitumor, and immunoregulatory activity. Noticeably, chitosan and its derivatives have been reported to act as potential antiviral therapeutic agents against coronavirus. Besides, agarose, a neutral marine carbohydrate extracted from red seaweeds *Rhodophyceae*, offers a variety of benefits, such as stimulating bone regeneration, wound healing, and neurogenesis [28]. Seven kinds of representative polysaccharide and the micro-nano nanosystems prepared based on them are listed below. Starting with chitosan, which is widely used and studied, we gave a detailed explanation of the material and shell requirements for chitosan-based nano/micro-materials in various drug delivery applications, including oral, intravenous, nasal, and local delivery. Besides, the advantages of chitosan itself, such as its biological activities, mucoadhesion, tight junction opening properties, cationic properties, etc., were highlighted. Additionally, the assembly processes, fundamental functions of nano/microparticulate assemblies, pharmacokinetics, pharmacodynamics, and their regulation on molecular pathophysiological processes in particular disease animal models of representative chitosan biomaterial-based nano/micro-systems were elaborated. This section also provided an overview of sodium alginate, including its gel-forming mechanism, physical and chemical characteristics, and biomedical applications. It also highlighted the advances in biomedical applications of chitosan or sodium alginate-based nano/micro-systems, as well as the current preclinical and clinical experimental studies, as well as the current challenges and future development directions in this field. Furthermore, the properties of the targeting effects (such as CD44 targeting and P-selectin targeting, respectively) of chondroitin sulfate-based and fucoidan-based nano/micro-materials as well as their potential applications in various diseases were described. This section also introduced a number of marine polysaccharides with gel-forming properties, such as ulvan, carrageenan, and agarose, and discussed their application as hydrogels.

### Chitosan-based nanomaterials

Chitosan, a unique, naturally occurring alkaline polysaccharide composed of varying numbers of (1–4)-glycosidic linkages between glucosamine and N-acetyl-d-glucosamine units, is obtained from renewable and natural marine sources by deacetylation of marine crustacean animal-derived chitin, the second most abundant carbohydrate on Earth after cellulose. The positive charge, adhesion properties, and mucosal adhesion properties confers advantages of chitosan as preferred vehicles for polyelectrolyte drugs (negatively charged substances) delivery as well as oral or nasal drug delivery [14]. And encouragingly the chitosan is Generally Recognized as Safe (GRAS) by the US Food and Drug Administration (FDA) which will undoubtedly assist in bridging the gap between the conceptual design of nanomaterials and their translation into actual commercial products for clinical use. The favorable biocompatibility of chitosan is applicable to the rational design of nano/micro-materials with reduced toxicity, decreased immunogenicity, and an ideal metabolic mechanism in vivo.

This section will elaborate on how the design of a chitosan-based drug delivery system may be fine-tuned in accordance with the varied properties of chitosan in order to achieve greater therapeutic effects.

#### Chitosan-based nanomaterials for enhanced oral drug delivery

Oral administration remains the preferred mode of drug delivery due to the ease of self-medication and favorable patient convenience, multiple dosage options, low skin irritation, and a large region of drug absorption in the gastrointestinal tract (GI). Orally administered drugs must overcome severe physiological obstacles, such as the intricate GI microenvironment (strongly acidic gastric fluid, bile salts, digestive enzymes, etc.) and the physical barriers (mucus layer and enterocyte), which, in combination with poor intestinal permeability and transintestinal transportation efficiency, limit the oral bioavailability of biological agents.

**Fig. 1:**
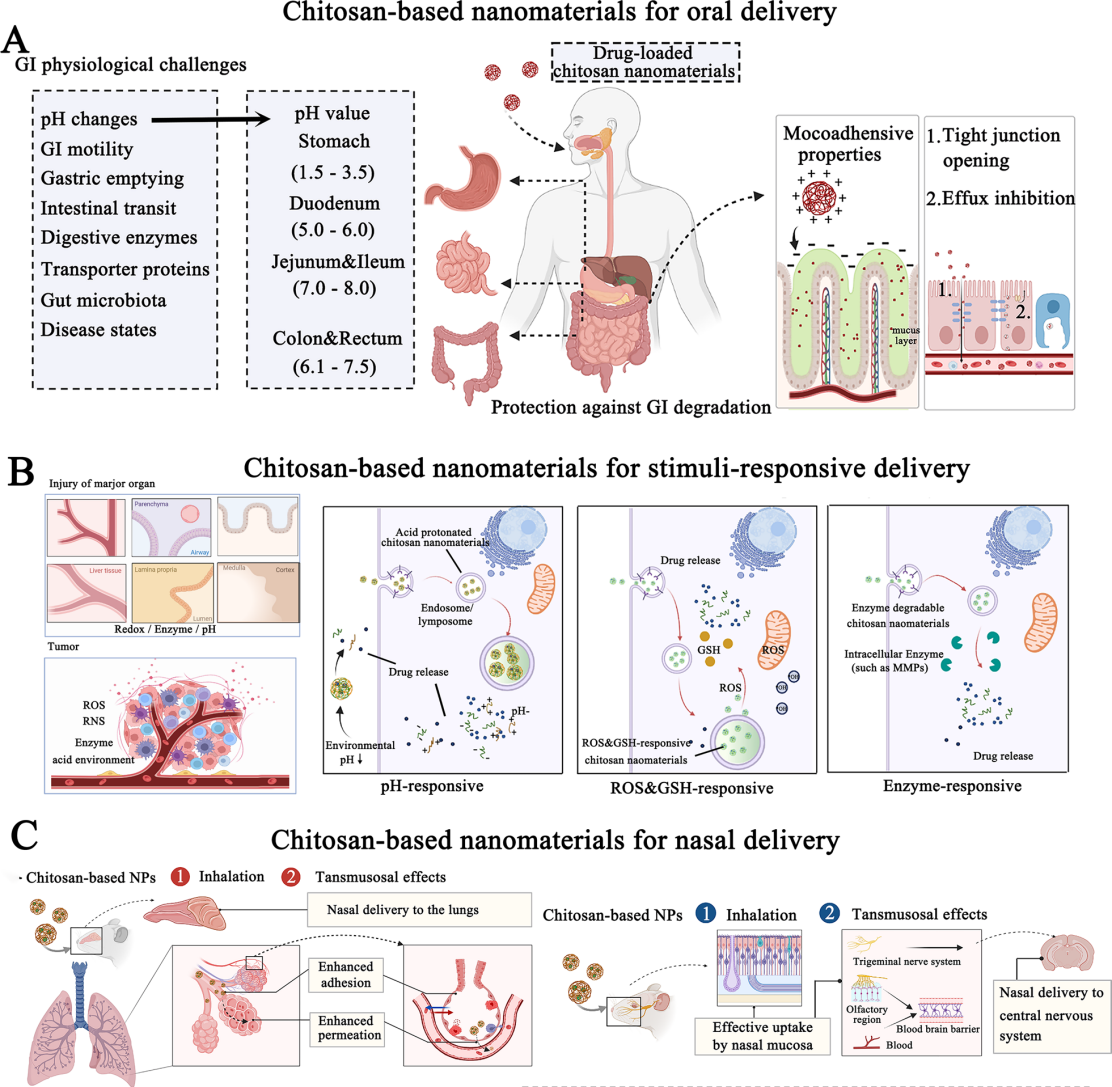
The schematic illustration of chitosan-based NPs for **A** oral drug delivery, **B** stimulus-responsive drug delivery, **C** and nasal delivery. Created with BioRender.com

Some of the characteristics of chitosan have contributed significantly to the widespread use of nanomaterials derived from chitosan as an oral dosage formulation: (1) Mucoadhesive property: the protonated amine groups endowed chitosan with a positive charge that could electrostatically interact with anionic counterparts present in the mucous layers, specifically sialic acid, to significantly enhance the mucoadhesive capacity. Besides, the functional amino groups of chitosan can form hydrogen bonds with glycoproteins in mucus, further resulting in a powerful adhesive effect. (2) Tight junctions (TJs) -opening capability: chitosan may interact with mucus and epithelial cells, causing a redistribution of cytoskeletal F-actin, the tight junction protein Zonula occludens-1 (ZO-1), and junctional adhesion molecule (JAM-1) translocation, resulting in instantaneous opening of cellular TJs and increasing payload intestine absorption via the paracellular transport pathway. Compared to the effects of recognized absorption enhancers, the effects of chitosan on the integrity of the tight junction appear reversible, and their effects on the integrity of the epithelium or cell membranes are negligible, making it more acceptable for serving as an oral dosage component [29]. (3) Adjusting drug release rate in the GI tract: chitosan's excellent compressibility enables it to be used in direct compression tablets with water-soluble or insoluble drugs. Assisting with gel and filming properties it could be used as a disintegrant for controlled release, or to accelerate dissolution. The hydrophilicity of chitosan (pKa: 6.3) in dilute acidic medium (usually in the acidic stomach) enabled the surface chitosan of tablets form a thick swelling gel layer for blocking the diffusion and dissolution of drugs. The release rate of hydrophilic drugs is determined by diffusion through the gel layer, whereas the release rate of hydrophobic payloads is increased by the gradual dissolution of the gel layer. Furthermore, chitosan degradation occurs in the colon due to the action of enzymes (such as lysozyme and other cellulases produced by colonic microorganisms) for sustained drug release. Figure 1A illustrates the mechanisms by which chitosan-based nanoparticles (NPs) enhance oral drug delivery. The advanced chitosan-based oral drug delivery systems will be discussed in this section.

1. Nanoparticles for oral small molecule drug delivery

Recently, chitosan or chitosan derivatives have been extensively used to be prepared as core–shell nanomaterials, and microcapsules for improving the payload stability, enhanced payload solubility, controlled drug release, and increased cellular uptake efficiency of a variety of hydrophobic agents or components (e.g., silymarin, curcumin, antibiotics, chemotherapy drugs camptothecin, etc.), thereby enhancing their oral bioavailability [16, 30–32]. Those design mainly focus on promoting the particle accumulation continuously in the mucosa due to chitosan adhesion effects and hence enhancing the bioavailability of drug for gastrointestinal diseases.

Besides, chitosan-based nanoplatforms have also been used in the treatment of other chronic diseases therapeutics via gastrointestinal administration, such as polycystic kidney disease (PKD). Metformin-loaded NPs (size: 150 nm, PDI: 0.24, surface charge: + 27.3 mV) were prepared for oral administration in chronic kidney disease by electrostatic interaction between chitosan, polyanion poly-L-glutamic acid, while the positively-charged payload metformin was easily incorporated during the ionic gelation process [33]. Orally administered to mice, there was a significantly higher accumulation of CS-based NPs (52.3%) in the intestines compared to the free drug (38.5%) at 48 h, and enhanced oral bioavailability (76.2%), resulting in a serum area under the curve (AUC) of NPs that was 1.3 times higher than that of the free drug in vivo. This study laid the groundwork for advancing chitosan nanotechnology for chronic diseases (requiring high dosages and repeated injections) beyond gastrointestinal disease, as well as resolving the key issue of systemic side effects caused by long-term intravenous injection of formulations exceeding the prescribed dosage.

To further improve the oral delivery efficiency of biomacromolecules, the following aspects could be taken into consideration:

(2) Nanopolyplex for oral protein/peptide delivery

**Fig. 2:**
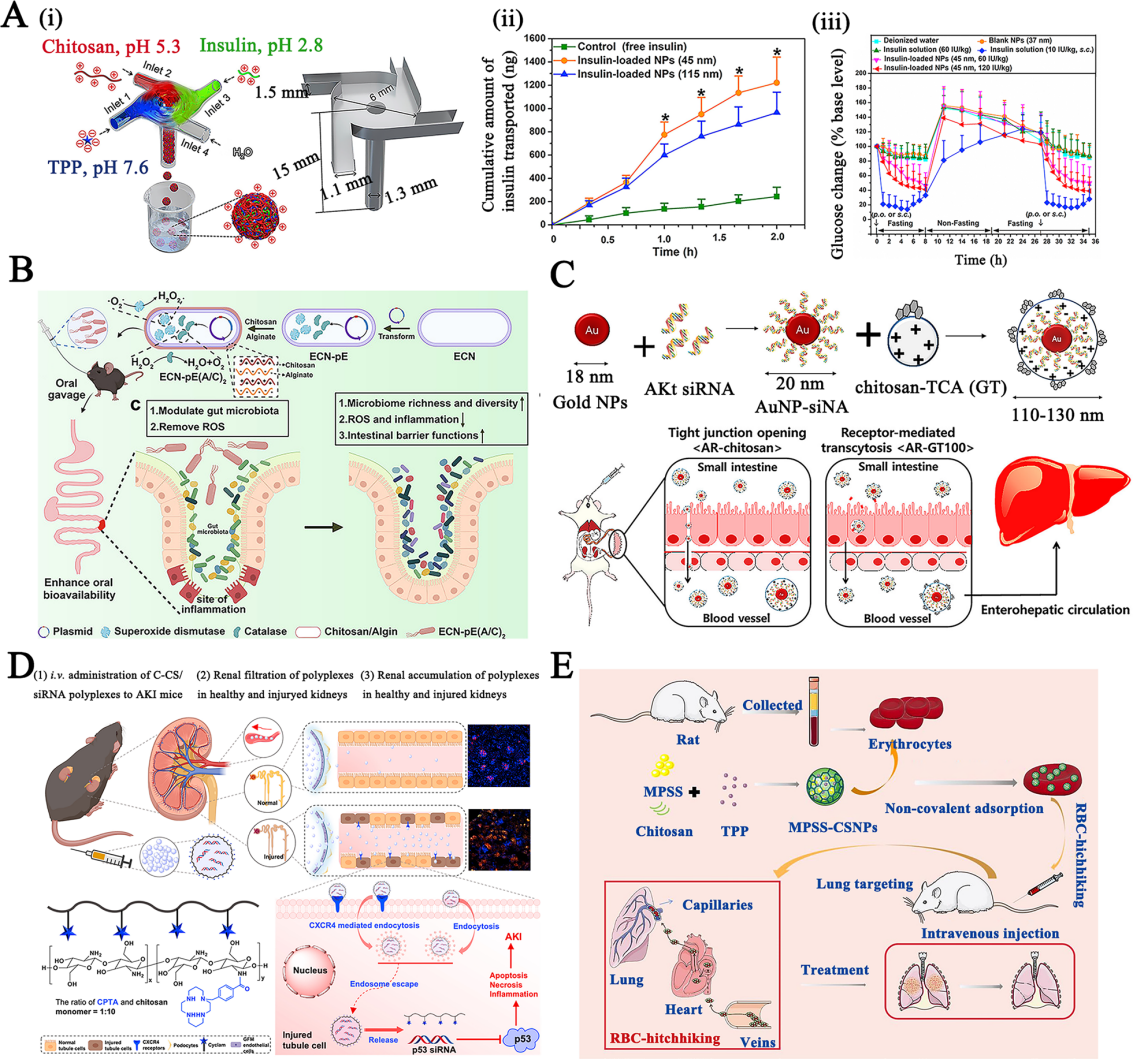
**A** (i) the preparation of chitosan/tripolyphosphate (TPP)/insulin nanocomplexes under kinetically controlled mixing; (ii) the effect of particle size on transepithelial transport across a Caco-2 monolayer; (iii) the blood glucose change curves of diabetic rats treated with different insulin formulations. Reproduced with permission [35]. Copyright 2017 Elsevier Ltd. **B** The preparation process of engineered probiotics encapsulated by chitosan and sodium alginate via the layer-by-layer strategy and the treatment mechanism in vivo; Reproduced with permission [43]. Copyright 2022 Springer Nature Limited **C** Schematic illustration of chitosan-taurocholic acid (GT) conjugate coated gold NPs and the drug delivery process. Reproduced with permission [57]. Copyright 2017 American Chemical Society **D** Schematic illustration of the structure of chitosan modified with α-cyclam-*p*-toluic acid (C-CS) and the treatment mechanism of the as-prepared C-CS/siRNA NPs. Reproduced with permission [71]. Copyright 2022 Elsevier Ltd. **E** The reparation of red blood cell (RBC)-hitchhiking methylprednisolone sodium succinate (MPSS)-loaded chitosan NPs and the treatment mechanism [76]. Copyright 2021 Elsevier B.V

The positive charge associated with the chitosan chains facilitates their interaction with neutral biomacromolecules (proteins, peptides) via ion–dipole interactions or electrostatic forces to form nanopolyplexes, which are the basis for the assembly of chitosan-based nanoparticles for the delivery of biomacromolecules, for high protein and peptide loading, while achieving sustained drug release [34]. He et al. utilized flash nanocomplexation techniques to prepare polyelectrolyte nanocomplexes by rapidly and efficiently infusing three components, including an aqueous solution of cationic chitosan, polyanion (tripolyphosphate, TPP), and anionic insulin, resulting in small size (45 nm) narrow size distribution (PDI: 0.14), highly colloidal stable, and a high encapsulation efficiency (90%) under kinetically controlled mixing conditions (Fig. 2A–i) [35]. Studies on transepithelial transport have demonstrated that insulin-loaded chitosan polyelectrolyte nanocomplexes with a size of 45 nm had superior adhesion and insulin transport across the mucus layer compared to NPs with a size of 115 nm, due to smaller nanoparticles diffusing to the apical surface of the epithelium more quickly than larger particles (Fig. 2A–ii). More importantly, insulin-loaded NPs had a better effect on blood glucose control in diabetic rats (dose produced a gradual but distinct reduction in blood glucose levels by about 50% within 8 h) than insulin alone (failed to induce any change in blood glucose level), achieving a ~ 10% oral bioavailability, further demonstrating the effectiveness of these insulin-loaded NPs (Fig. 2A–iii).

(3) Microcapsules for oral protein/peptide delivery

Chitosan could form microcapsules with anionic polysaccharide alginate due to strong interactions between the amino moieties of chitosan and the carboxylic groups of alginates, yielding polyelectrolyte complex with pH-sensitive swelling properties for enhancing stability in acidic gastric juice as their interaction could be easily broken down and releasing payloads in the intestine [36–39]. Yang et al. proposed a novel postbiotic synergistic delivery microcapsule formed by the restriction of indole‐3‐propionic acid (IPA) drug in a resistant starch (RS) and solidified crosslink structure between Ca^2+^ and alginate via a capillary microfluidic electrospray method, followed by coating with chitosan via spontaneous electrostatic interactions (between chitosan and alginate), which minimizing the leakage of IPA agents [40]. The optimal particles exhibited a 200 nm diameter, a wrinkled spherical morphology, and an exceptionally rough surface by varying the microfluidic flow rate and electrospray voltage. Compared to the smooth particles, the wrinkled microcapsules had a stronger adhesion ability and a prolonged intestine retention duration of 12 h after oral administration, resulting in significant protective effects against colitis, including lower inflammation levels in colonic tissues and a large increase in the total diversity and abundance of beneficial bacteria.

In addition, chitosan and alginate can be assembled layer by layer to achieve colon-specific delivery [41, 42]. In another study, innovative oral chitosan/alginate capsules based on the layer-by-layer electrostatic self-assembly of chitosan and sodium alginate were produced to provide protective effects against encapsulated engineered antioxidant probiotic ECN-pE (Fig. 2B) [43]. Because the outer alginate coating shrinks and forms a skin-like structure to block acid and bile salts, as well as the potent mucosal adhesive capability of the chitosan layer, the chitosan/alginate coating exhibited exceptional gastrointestinal tolerance, resulting in a fourfold and 1.5-folds increase in the number of living ECN-pE in the stomach compared to that of uncoated ECN-pE and clinically used enteric coating (Eudragit L100-55), respectively. In comparison with the control group, the formulation administered orally obviously protected mice from DSS-induced colitis, including body weight loss, shortened colon length, and damage to colonic tissue.

Besides, chitosan tablets or microcapsules could also be typically coated with several pH-responsive polymers, such as polyacrylic polymers (Eudragit S100 or L100117), hydroxypropyl methylcellulose phthalate (HPMCP), etc., for preventing the protein/peptide release in the stomach but permitting their release in the intestine due to the dissolution of coating materials at higher intestinal pH [44, 45].

(4) Mucus penetrating and mucoadhesive NPs for oral protein/peptide delivery

It should be noted that only soluble and protonated chitosan (cationic properties) can stimulate the opening of the tight junctions, thus promoting the paracellular transport of compounds, implying that chitosan with poor solubility have limited mucoadhesive characteristics and can only be effective as an absorption enhancer in a limited area of the intestinal lumen where the pH values are close to its pKa. Encouragingly, several chitosan derivatives (generally modified on the abundant amino and hydroxyl moieties of the chitosan backbones) with improved solubility at a wider pH range were fully utilized in the development of advanced materials for drug delivery, with these derivatives including carboxymethyl chitosan, glycol chitosan, trimethyl chitosan (TMC), N-succinyl-chitosan, thiolated chitosan, hydroxypropyl trimethyl ammonium chloride chitosan (HACC), sulfated chitosan, and among others [29, 46, 47]. In addition, thiolation on the chitosan (such as chitosan-cysteine, chitosan-glutathione, chitosan-thioglycolicacid, chitosan-N-acetyl cysteine, etc.) is a typical strategy for increasing the mucoadhesive capability due to the production of disulfide bonds between the thiol group of the polymer and cysteine-rich subdomains of mucus glycoproteins.

Nevertheless, the mucoadhesive property of chitosan materials may have a minimal effect on particle penetration over the mucosal barrier and these NPs are easy to be eliminated by natural mucus turnover [24, 48]. To solve the problem, chitosan could be chemically modified with penetrating peptides (like protamine, D-octaarginine, L-penetratin, and others) or mucopenetration-enhanced polymers to synergistically boost the oral bioavailability of biomacromolecules [49, 50]. Besides, the penetrating effects of chitosan NPs might be improved upon altered physiochemical characteristics of carriers, such as optimized small size (< 100 nm), enhanced hydrophilicity (for enhancing solubility), and surface charge shielding (net-neutral surfaces diffused more efficiently through the mucus layer) [24, 43, 51–55].

(5) Active targeting NPs for oral protein/peptide delivery

Several targeting ligands, like essential nutrients (such as vitamins B12), glycans (mannose, galactose, and hyaluronic acid), transferrin, and hormones, could be extensively explored for enhancing active transport of chitosan-based NPs via receptor-mediated endocytosis for facilitating the oral absorption of biomacromolecules [44]. For example, Jiang et al. designed atorvastatin- and galactose-modified TMC NPs (size: 150 nm, surface charge: + 20 mV) encapsulated with siRNA (miR-33, which plays a critical role in regulating inflammation) for dual targeting to hepatocytes and macrophages via receptor-mediated endocytosis, achieving efficient gene silencing that outperformed commercial gene translation agents, Lipo-8000 [56]. The particles were able to preserve the payload gene drug from degradation in body fluid for 4 h, which necessitated significant accumulation of the gene drug in the target tissues.

In contrast to the use of receptor-mediated endocytosis, it is another feasible strategy to increase oral bioavailability by modifying chitosan with compounds that are preferentially conveyed by the transporters on the surface of epithelia in order to enhance the absorption of intestinal enterocytes and target tissues via the enterohepatic circulation pathway. For example, negatively charged gold NPs wrapped by positively charged glycol chitosan-taurocholic acid (GT) conjugate (yielding NPs with an average size of 110–130 nm and a surface charge of + 29 mV) are able to enhance the oral absorption of the loaded thiolated Akt siRNA through bile acid-mediated transport of apical sodium bile acid transporters (ASBT) on the intestinal enterocytes (Fig. 2C) [57]. After oral administration, GT conjugated NPs exhibited an approximately 1.2–1.4 times increase in accumulation in both ileal tissue and the liver compared to the sole chitosan NPs at 6 or 12 h, respectively. Therefore, the formulation promoted Akt gene silencing, induced colorectal liver metastases cell apoptosis, and ultimately enhanced gene therapy efficacy in a mouse model of colorectal liver metastases cancer.

(6) Polymer/gene complex for enhancing transfection efficiency

The amino groups of chitosan contribute to the complex’s escape from the endosome by triggering a “proton sponge effect” similar to that of traditional jetPEI or Lipofectamine^®^ (Lipo-2000 or Lipo-3000) series of transfection reagents, but comfortingly, chitosan itself maintains a higher biosafety than these traditional transfection reagents [58]. These properties further broadened its applications for oral delivery of gene drugs.

However, in comparison to other commercial transfection reagents (e.g., jetPEI, Lipo), the transfection efficiency of chitosan is mediocre. For improving the transfection efficiency of gene drug loaded chitosan NPs, a gene delivery vehicle composed of pDNA and chitosan-grafted branched polyethyleneimine (bPEI, low Mw: 0.8 kDa) with a variety of grafting degrees of substitution (DS) of bPEI (0% ~ 70%) has been described in a recent study [59]. The increased DS of PEI endowed NPs with a more compact structure and significantly enhanced the protective effects of insulin-pDNA against acidic denaturation and enzymatic degradation, a prerequisite for efficient systemic cell transfection in vivo. The gene translation efficiency increased by as much as 7.5‐fold, outperforming 25 kDa bPEI in terms of gene expression. Besides, repeated three‐time dosing did not induce potential toxicity in the parenchymal organs of mice. Nevertheless, the high grafting density of PEI on the chitosan backbone (which may bind the shorter siRNA or miRNA more tightly) must be further optimized to produce more favorable intracellular unpacking kinetics of gene therapeutics.

In addition to the aforementioned exemplary examples of chitosan-based NPs for oral drug delivery, Tables 1, 2 provides an overview of other recent chitosan-based nanomaterials for oral small molecular drug and biomacromolecules delivery.

**Table 1:** Chitosan-based nanomaterials for oral delivery

Type of nanomaterials	Properties of chitosan	Payload	Assembly mechanism or preparation method	Characterization	Mechanism	Animal model	Oral bioavailability	Refs.
mPEG-chitosan-oleic acid micelles	Mw: 112 kDa, DD: 15%	CPT	Self-assembly	Size: 178 nm; PDI: 0.28; Surface charge: + 42.8 mV; EE: 55.5%, LC: 8.3%	Chitosan-mediated mucoadhesive effects	Chemically induced colorectal cancer model	10% (24 h)	[30]
Gemcitabine-loaded CSKSSDYQC (CSK)-TMC NPs	Mw: 400 kDa DD: > 90%	Gemcitabine	Self-assembly	Size: 173.6 nm; PDI: 0.2; Surface charge: + 18.5 mV; EE: 66.4%, LC:19.4%	CSK-mediated goblet cells active targeting	4T1 breast tumor mice model	60.1%	[139, 236]
Caseinate/triphenylphosphonium -chitosan/alginate NPs	DD: 95%, Viscosity: 100–200 mPa·s	Caseinate	Polyelectrolyte complexation	Size: 430 nm; EE: 75.3%, LC: 5.2%	Alginate mediated pH responsiveness; triphenylphosphonium-mediated mitochondrial targeting	DSS-induced colon colitis mice model	–	[16]
Chitosan/ 4-(hydroxymethyl) phenylboronic acid pinacol ester (PAPE)-modified fucoidan NPs	–	Small molecule drugs: Phein	Polyelectrolyte complexation	Size: 233.1 nm; PDI: 0.15; EE: 93.6%	(1) pH-responsiveness (2) Enzymatic degradation of chitosan in colon; (3) PAPE-mediated ROS-responsiveness	DSS-induced colon colitis mice model	–	[237]
Chitosan/poly-L-glutamic acid NPs	Mw: 150 kDa, DD: 95%	Metformin	Ionotropic gelation	Size: 150 nm; PDI: 0.24; Surface charge: + 27.3 mV	pH-responsiveness	Polycystic kidney disease mice model	–	[33]
Chitosan-binding peptide (CP)/PEG-DSPE/PLGA NPs	–	Itraconazole	Surface coating	Size: 136 nm; PDI: 0.24; Surface charge: + 21.5 mV	(1) 12-mer peptide (ADGVGDAESRTR)-mediated targeting (2) mucus adhesion and pH-responsiveness	*C. neoformans*-infected mouse models	–	[29]
CUR-loaded PVA/ guar gum NPs coated with alginate/chitosan microgels	Mw: 1000 kDa DD: 95%	CUR	Polyelectrolyte interactions	Size: 400 μm; EE: 43.8%; LC: 16.1	(1) Colon enzymatic degradation (2) pH-responsive swelling of the inner layer	DSS-induced colon colitis mice model	–	[238]
Vancomycin-loaded chitosan-polyaniline microgels	Mw: 190–310 kDa	Vancomycin	Emulsion method	Size: 243.1 nm; PDI: 0.15; EE: 91.3%	lysozyme-cleavable 1,4-β-glycosidic bonds of chitosan for drug release	–	–	[239]
Resveratrol-loaded *Antheraea pernyi* silk fibroin NPs embed in chitosan-alginate hydrogels	–	Resveratrol	Iron crosslinking	EE: 68.2%, LC: 6.2%	(1) pH/ROS/GSH- responsiveness (2) Integrin receptors-targeting in colon	DSS-induced colon colitis mice model	–	[53]
AC-BSA coated with glycol-chitosan and EGAC (organic–inorganic hybrid nanocomposite)	–	BSA	Sequentially surface coated	size: 325 nm; PDI: 0.35; surface charge: − 33.2 mV; EE: 100%;	Layer-by-layer deposition enhanced stability and intestinal permeation	Normal rat model	–	[240]
Chitosan/insulin-loaded zein-carboxymethylated short-chain amylose nanocomposites	Mw: 140 kDa, DD: 90%	Insulin	Surface coated with chitosan	Size: 311 nm; PDI: 0.22; Surface charge: + 43.7 mV; EE: 89.0%, LC: 6.8%	Chitosan as a permeation enhancer	Diabetic rat model	15.1%	[241]
Insulin/HTCC-chitosan complex coated with thiolated hyaluronic acid (core–shell NPs)	Mw: 50 kDa, DD: 95%	Insulin	Polyelectrolyte complexation (based FNC)	Size: 102 nm; PDI: 0.11; Surface charge: − 26.2 mV EE: 91.0%, LC:38.0%	(1) HTCC-chitosan with enhanced solubility (2) HA-SH enhanced mucus-penetration	Type 1 diabetic rats	11.3%	[54]
Alginate/chitosan microparticles	–	AvrA protein	Iron crosslinking (Ca^2+^)	Size: 281 nm; PDI: 0.32; Surface charge: − 11.6 mV; EE: 89.0%, LC: 6.8%	pH-responsiveness for inflammatory colon-targeting	DSS-induced colitis mice model	1%	[51]
Insulin-loaded deoxycholic acid modified chitosan NPs	Mw: 100 kDa, DD: 90%	Insulin	Polyelectrolyte complexation	Size: 226.1 nm; PDI: 0.18; Surface charge: + 14.3 mV; EE: 73.5%, LC: 33%	Deoxycholic acid promoted NPs traverse the intestinal epithelium by exploiting the bile acid pathway	Streptozotocin-induced diabetic rats model	15.9%	[242]
Chitosan/TPP/insulin NPs	Mw: 90 kDa, DD: 85%	Insulin	Polyelectrolyte complexation based on FNC	Size: 45 nm; PDI: 0.14; Surface charge: + 9.4 mV; EE: 91.0%, LC:27.5%	The smaller size NPs (45 nm) exhibited better hypoglycemic effects over large size NPs (115 nm)	Streptozotocin-induced diabetic rats model	–	[35]
Chitosan-g-bPEI/pDNA NPs	Mw: 15 kDa, DD: ~ 85%	Insulin-pDNA	Polyelectrolyte complexation	Size: ~ 160 nm; PDI: 0.32; Surface charge: + 37 mV	(1) PEI (0.8 kDa) conjugation enhanced transfection efficiency and reduce toxicity (2) Prolonged the intestinal retention	STZ‐induced diabetic mice model	–	[243]
H6P/arginylglycylaspartic acid and mannose-modified chitosan NPs	–	Heat shock protein (H6P)	Polyelectrolyte complexation	Size: ~ 320 nm	M cell targeting (RGD peptide) DC cell targeting	NOD mice	–	[244]
Fluorocarbon-modified chitosan/ antibodies capsules	–	αPDL1 antibody	Polyelectrolyte complexation	Size: ~ 100 nm Surface charge: + 15 mV	Fluorocarbon chains with hydrophobic and lipophobic behaviors enhance cross-membrane penetration	C57BL/6 mice bearing B16F10 melanoma tumors	4.7%	[245, 246]
siRNA/mannose-modified trimethyl chitosan-cysteine/TPP-based NPs	TMC Mw: 200 kDa, DD: 85%	siRNA	Polyelectrolyte complexation	Size: ~ 150 nm; PDI: ~ 0.2; Surface charge: + 18.7 mV	Mannose-mediated targeting (to macrophage) caveolae-mediated endocytosis for robust siRNA delivery	LPS/D-gal induced acute liver injury	–	[247–250]
Chitosan coated siRNA-loaded lanthanum phosphate nanoparticles (CS/LaP/siRNA NPs)	–	SiRNA LAP	Polyelectrolyte complexation	Size: 210 nm; Surface charge: + 27.0 mV	Chitosan was used as the outer shell to control the excessive growth of lanthanum phosphate complexes	Colorectal cancer mouse model	–	[251]
Glycol chitosan–taurocholic acid (GT) coated AuNP–siRNA nanocomplex	Mw: 82 kDa	Akt2-siRNA	Polyelectrolyte complexation	Size: 100 ~ 130 nm Surface charge: + 0.4 mV	(1) GT forms a protected layer (2) Taurocholic acid moiety targeting apical sodium bile acid transporters receptor	Orthotopic colorectal liver metastases mice model	–	[57]
Oxaliplatin and siRNA/folic acid-conjugated CS NPs embed in chitosan and alginate layer-by-layer (LbL) film	Mw: 100–300 kDa DD: 75–85%	Oxaliplatin and siRNA	Polyelectrolyte complexation	Size: 238 nm; Surface charge: + 27.1 mV; EE: > 90%	Folic acid receptor -mediated tumor targeting delivery	Azoxymethane and DSS-induced colon cancer mice model	–	[252]
siRNA/mannose-modified TMC/anionic crosslinkers (TPP, ES, and HA) NPs	Mw: 200 kDa, quarternization degree: 30%	siRNA	Ionic gelation	Size: 150–200 nm; PDI: 0.1–0.17; surface Charge: + 18.9 ~ + 37.0 mV	Mannose-mediated macrophage targeting different anionic crosslinkers mediated different cellular unpacking kinetics	Acute hepatic injury mice model	–	[253]
OPBP-1 loaded TMC hydrogel	–	OPBP-1	–	Swelling degree of the hydrogel reached 94.3% at 1 h	Protected the payloads from the protease degradation	CT26 tumor mice model	52.8%	[47]
Gal-siTNF-PLGA NPs loaded alginate/chitosan hydrogel	Mw: 18 kDa	siTNF	Ionic gelation	–	Alginate/chitosan specific degradation in inflamed colon for release payloads	DSS-induced colitis mice model	–	[37, 51]

*Mw* molecular weight, *DD* degree of deacetylation, *EE* encapsulation efficiency, *LC* drug loading capacity, *CUR* curcumin, *SN387*-ethyl-10-hydroxycamptothecin, *DSS* dextran sodium sulfate, *STZ* streptozotocin, *UC* Ulcerative colitis , *DOX* doxorubicin, *CPT* camptothecin, *PTX* paclitaxel, *TMC* N,N,N-trimethyl chitosan, *TPP* tripolyphosphate, *BrijS20* Polyethylene glycol octadecyl ethe, *Pgp* P-glycoprotein, *TJs* tight junctions, *FNC* flash nanocomplexation, *PBCA* poly (n-butylcyanoacrylate), *BSA* bovine serum albumin, *AC* 3-aminopropyl functionalized magnesium phyllosilicate, *EGAC* Eudragit^®^L100–55, *siRNA* small interfering RNA, *HTCC* N-(2-hydroxypropyl)-3-trimethyl ammonium chloride modified chitosan, *LAP* lanthanum phosphate, *HA* hyaluronic acid, *Eudragit*^*®*^* S100, ES* methylacrylic acid-methyl methacrylate copolymer, *OPBP-1* Oral PD-L1 Binding Peptide 1

**Table 2:** Chitosan-based nanomaterials for systemic administration

Type of nanomaterials	Properties of chitosan	Payload	Type of NPs targeting	Assembly mechanism or preparation method	Characterization	Targeting mechanism or drug release mechanism	Animal model	Refs.
siRNA-loaded chitosan-lactate NPs	DD: 87.7%	Anti-CTLA-4	Passive targeting	Polyelectrolyte complexation	size: 77 nm; PDI: ~ 0.2; surface charge: + 14 mV;	Tumor targeting via EPR effects	CT26, and 4 T1 cell xenograft mice model	[254]
PTX-loaded chitosan–polyethylene glycol nanofiber	Mw: 3.9 kDa	PTX	Passive targeting	Chemical covalent linkage	1) Nanofiber: size: 565 nm; surface charge: + 0.8 mV; 2) smaller NPs: size: 20.6 nm; surface charge: -5.8 mV	(1) Nanofiber break down into smaller NPs by interaction with serum proteins; (2) Tumor targeting via EPR effects	Aggressive and drug-resistant breast cancer and melanom mouse models	[63]
miRNA mimics/TPP/PEG-chitosan NPs	Mw:50 ~ 190 kDa DD: 75 ~ 85%	miRNA (miR-33)	Active targeting	Polyelectrolyte complexation	Size: 150 ~ 200 nm; Surface Charge: + 2 ~ + 6 mV	(1) Naïve macrophages-targeting; (2) ABCA1 gene silencing for regulating the cholesterol efflux	acLDL-loaded/[3H]cholesterol-labeled peritoneal macrophages injected mice model	[68]
siRNA/α-cyclam-p-toluic acid (CPTA)-modified chitosan NPs	DD: 85% Mw: 56 kDa CPTA: chitosan = 1:10	p53-siRNA	Active targeting	Polyelectrolyte complexation	Size: 129.5 nm; PDI: 0.23; Surface charge: + 14.8 mV	(1) CPTA-CS targeted renal CXCR4 receptor (2) Chitosan targeting proximal tubule cellular surfaces	Ischemia–reperfusion injury (I/R) induced acute kidney injury (AKI) mice model	[71]
Mesoporous silica shell/POM nanoclusters coated with chitosan-FA	Mw: 60 kDa	DOX	Active targeting	electrostatic interaction	size: ~ 120 nm; surface charge: − 10.5 mV	FA targeting FA receptor in tumor cells	U14 cell xenograft mice model	[61]
SS-31/HA/chitosan NPs	–	SS-31	Active targeting pH-responsive	Polyelectrolyte complexation	Size: 53 nm; PDI: 0.2; Surface charge: − 19.6 mV; EE: 94.0%, LC: 10.5%	(1) CD44-targeting (HA), mitochondria-targeting (SS31) (2) pH-responsive releasing	I/R-induced AKI mice model	[88]
siRNA/ HA dialdehyde/chitosan	Low Mw: 29 kDa DD: 93.7%	Bcl-2 siRNA	Active targeting	Polyelectrolyte complexation	size: 100 ~ 120 nm; PDI: ~ 0.1	CD44-targeting (HA)	T24 tumor cell xenograft mice model	[255]
SiRNA/PEG/ mannose modifed-TMC/ PC NPs	TMC Mw: 200 kDa DD: 85%	VEGF siRNA/PIGF siRNA	Active targeting pH-responsive	Polyelectrolyte complexation	Size: 144 nm; PDI: 0.14; surface Charge: + 15.7 mV	Acidic-responsive benzimide bond cleavage of PC mannose-mediated active-targeting	In situ and lung metastatic breast cancer models	[256]
Red blood cell (RBC)-hitchhiking drug/TPP/chitosan NPs	DD:85%	Methylprednisolone sodium succinate	Active targeting	Ionotropic-gelation method	Size: 233 nm; Surface Charge: + 30 mV; EE: 80%	RBC-hitchhiking for lung targeting	LPS-induced acute lung injury	[76]
CPP-chitosan-co-PNVCL core/shell NPs	Mw: 10 kDa DD: > 95%	DOX	pH-responsive	Self-assembly of amphiphilic polymer	Size: 166 nm; PDI: < 0.45; Surface Charge: + 15.4 mV; EE: 85.3%, LC: 14.8%	(1) The amide bond between CPP and chitosan cleaved by the MMPs (2) Chitosan-mediated acidic-responsive drug release	MCF-7 tumor-bearing xenograft mice	[257]
Chitosan-octenylsuccinic anhydride	O-carboxymethyl chitosan 12 mPa·s	γ-Fe_2_O_3_/isosorbide dinitrate	pH-responsive	Self-assembly of amphiphilic polymer	Size: 150 ~ 180 nm	Chitosan-mediated acidic-responsive drug release	H22 hepatoma cell-bearing tumor model	[66]
Chitosan/alginate hydrogel	DD: 87 ~ 90%	Cisplatin (CDDP) and DOX	pH-responsive	Cross-linking	(DOX), EE: 83.0%, LC:86.0%; (CDDP), EE: 84.0%, LC:81.0%	Alginate-mediated swelling and chitosan-mediated acidic-responsiveness	–	[82]
TH-302 loaded chitosan-bilirubin NPs	Mw: 3 kDa	Hypoxia-activated prodrug (TH-302)	ROS-responsive	Self-assembly of amphiphilic polymer	Size: 116 nm; EE: 75%	ROS-responsive hydrophobic bilirubin converted into biliverdin with improved aqueous solubility	HeLa tumor-bearing mice model	[90]
l-serine–modified chitosan-TK-SS31 NPs	Mw: ~ 2.5 kDa	SS31	ROS-responsive active targeting	–	–	(1) l-serine targeting to kidney injury molecule–1 (Kim-1) in kidney tubule (2) TK bond mediated ROS-responsiveness	I/R-induced AKI mice model	[258]
Pazopanib-FA-chitosan-TK hydrogel	Mw: 150 kDa	Pazopanib AQ4N	ROS-responsive	In situ formation of hydrogel with the effects of enzyme (Laccase)	–	(1) Enzyme-mediated dimerization of FA to achieve oxygen-triggered gelation (2) TK-mediated ROS-responsiveness	4T1 mouse breast tumor model	[92]
PEI-ss-HECS-ss-OA micelle coated with HA	Mw: 100 kDa DD: 90%	siRNA PTX	GSH-responsive enzyme-responsive active targeting	Self-assembly of amphiphilic polymer	Size: 194 nm; PDI: 0.21; surface Charge: − 21.3 mV	(1) HA-mediated CD44 receptor targeting (2) enzyme-responsive (HAase) (3) GSH-responsive (disulfide bond)	BALB/c nude mice bearing A549 lung cancer	[84]

*CXCR4* C-X-C chemokine receptor type 4, *CTLA-4* Cytotoxic T-Lymphocyte–Associated Antigen 4, 2,2,6,6-tetramethylpiperidine 1-oxyl (TEMPO), *FA* folic acid, *TMC* trimethyl chitosan, citraconic anhydride grafted *PC* poly allylamine hydrochloride, *PNVCL* poly(N-vinylcaprolactam), *DOX* doxorubicin, *TK* thioketal bond

Overall, chitosan-based particles have been proven to be promising carriers for enhancing the oral bioavailability and bioactivity of a wide range of drugs by specifically overcoming systemic physicochemical barriers.

#### Chitosan-based nanomaterials for intravenously targeting drug delivery

Targeted drug delivery often entails the precise delivery of payloads to the lesion by an active or passive strategy, thereby effectively targeting the desired biological sites (e.g., multiple types of cancer cells, inflammatory tissues, etc.). In this section, we will discuss in depth the advanced development of targeted chitosan-based nanomaterials for intravenous administration. Table 2 provides examples of typical chitosan-based NPs with targeting capabilities.

As for passive targeting, several chitosan NPs with particle sizes within 10 ~ 200 nm or cationic surface properties trend to be accumulated around tumor sites via enhanced permeability and retention effects (EPR effects) or possessing a strong affinity for negatively charged membranes (like tumor and bacterial membranes) for enhancing the targeting effects [34, 60–62].

Nevertheless, small-size NPs often have a restricted drug loading capacity, and positively charged chitosan NPs are growing in size due to their susceptibility to interact with serum proteins, leading to poor colloidal stability. Zhang et al. present a strategy for fabricating CHI-PEG-PTX nanofibers via a self-assembly process of a conjugate that was synthesized by chemically linking polyethylene glycol (PEG) and 2′-succinyl paclitaxel (PTX) to the abundant primary amine groups of chitosan [63]. Once entry into the circulation, electrostatic interactions between protein and chitosan as well as the hydrophobic interactions between PTX and serum albumin may disrupt the intermolecular hydrogen bonding of the fibrous structures, resulting in the in situ fragmentation of nanofibers and decomposition into smaller-size nanocarriers (20.6 nm). The approach achieved a high drug loading capacity (8.4 wt% of PTX), while preserving colloidal stability and a reduced size in the blood, resulting in a prolonged circulation time. Most of the smaller-sized decomposed NPs accumulated in tumors 24 h after systemic injections due to EPR effects, providing high potency in inhibiting tumor growth (by more than 75% inhibition rate in mouse xenograft melanoma model) and metastasis (by 66% inhibition rate in xenograft mouse).

As a result of unfavorable interactions with the components of biological fluids and insufficient efficacy in reaching the specific sites (such as the cytosol and nucleus) of targeted cells, passive targeting cannot always guarantee effective treatment outcomes. In terms of active targeting, there are typically three cases:

1. Chitosan, chitosan oligomers, and several chitosan conjugates tend to accumulate in the kidneys due to the reactive amino groups and glucosamine units that might trigger megalin receptor-mediated internalization into renal proximal tubule cells, thereby contributing to the renal-targeted delivery of payloads (chitosan-mediated organ-targeting) [64, 65]. Additionally, chitosan could bind with glycoprotein receptors expressed on blood cells (such as monocytes, macrophages, and dendritic cells, etc.), resulting in the selective internalization of vehicles (chitosan-mediated cell-targeting) [66, 67]. Utilizing the glycoprotein receptor-targeting effects of chitosan, Nguyen et al. developed macrophage-targeting functional chitosan NPs (size: 150–200 nm) by the complexation of PEGylation chitosan, TPP, and negative miRNA mimics (miR-33 that could positively regulate cholesterol efflux) via the ionic gelation process [68]. Upon naïve macrophages cell model, the NPs exhibited a rapid uptake into the cytosol of naïve macrophages via endocytosis within 8 h of treatment and escape from the endosomes and release their cargos into the cytosol in a timely manner, thereby promoting the miR-33-mediated cholesterol efflux from macrophages (an essential step in reverse cholesterol transport) and lipid removal from foam cells.
2. Chitosan NPs could also be modified with targeting ligands such as folic acid (FA), hyaluronic acid (HA), protamine, arginylglycylaspartic acid (RGD), phenylboronic acid, lactose acid, nucleolin-targeting aptamers, and others to enhance the organ/tissue targeting ability and cellular uptake after systemic administration [69, 70]. Tang et al., for example, reported an electrostatically complexed renal-targeted delivery system (size: 129 nm, surface charge: + 14.8 mV) formed by α-cyclam-p-toluic acid (CPTA)-modified chitosan and siRNA (Fig. 2D) [71]. The modified CPTA-chitosan has antagonistic and targeting properties toward the chemokine receptor CXCR4 overexpressed in injured kidney cells, but the parent CPTA lacked these properties. The accumulation of NPs in the lesion of injured kidneys was ~ 2.5 times higher than in normal kidneys, resulting in significant restoration of renal function, alleviation of cell apoptosis, and inhibition of macrophage and neutrophil infiltration, thus providing an innovative strategy for the treatment of acute kidney injury (AKI). However, it is necessary to optimize the size of these CPTA-modified chitosan NPs for achieving smaller sized particles as carriers smaller than 100 nm have superior accumulation in the kidney, for further improving their renal-preferred location [72].
3. It is worth noting that several studies have reported an intriguing breakthrough in the development of biomimetic chitosan-based active targeted drug delivery systems by coating anionic cell membranes through electrostatic interactions [73]. Ding et al. prepared red blood cell-hitchhiking chitosan NPs (prepared by ionic crosslinking of chitosan/TPP/methylprednisolone sodium succinate drug) to avoid the rapid removal from the bloodstream by mononuclear phagocyte systems and to improve targeting efficiency toward endothelium-rich lungs (Fig. 2E) [74–76]. Due to the prolonged in vivo circulation time and substantial accumulation at the lesion, the *i.v.* injection of red blood cell (RBC)-hitchhiking NPs significantly reduced the pro-inflammatory cytokine levels within 48 h in the lipopolysaccharide (LPS)–induced acute lung injury mouse model. Note that the relationship between physicochemical properties (surface charge, hydrophilicity/hydrophobicity, and particle size) of chitosan NPs and affinity toward cell membranes has not yet been elucidated with sufficient precision for targeting different organs or at least differentiating pathological from non-pathological tissues [73].

#### Chitosan-based stimuli-responsive delivery systems for on-demand drug delivery

The chitosan could be modified with sensitive chemical bonds, conjugates, etc. as intelligent microenvironment-responsive drug delivery systems to achieve the enrichment, penetration, and responsive delivery of encapsulated drugs to desired sites in response to abnormal indicators of extracellular compartments such as pH change, hypoxia (or energy change), oxidative metabolism disorder (such as high reactive oxygen species (ROS) and glutathione (GSH)), overexpression of enzymes, aberrant metabolism of amino acids, and incomplete vascular structures, etc. (Fig. 1B) [77–81]. Table 2 summarizes the related assembly/disassembly process and mechanism of these “smart” chitosan-based formulations, targeting mechanism, properties of the chitosan-based particles, and drug release behavior of chitosan-based NPs over the past three years. In this section, the chitosan carriers with stimulus-responsive properties are illustrated.

1. pH-responsive chitosan NPs

pH gradients often exist among subcellular substructures or pathological microenvironments, such as ischemia, infections, inflammation, tumors, etc., which are generally more acidic than normal tissues [80]. These are the strategies used for pH-responsive drug release from chitosan NPs: (1) Chitosan networks swell under acidic circumstances and shrink under neutral or alkaline conditions. Therefore, non-interacting drugs are rapidly released in an acidic environment [82]. (2) The cleavage of pH-responsive bonds (e.g., hydrazones, ester bonds) or the elimination of pH-sensitive groups may lead to the disassembly of carriers [83–87].

**Fig. 3:**
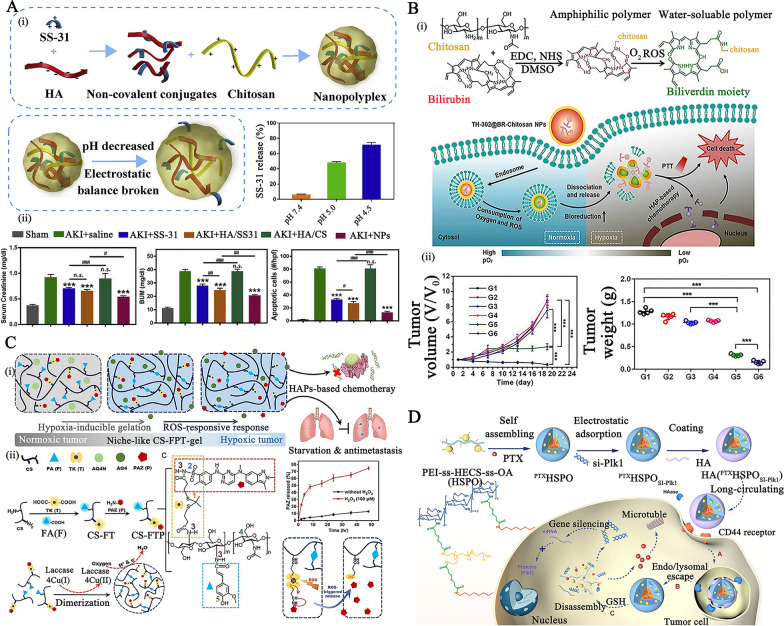
**A** (i) schematic illustration of the hyaluronic acid (HA)/chitosan/SS31 NPs assembly process; (ii) the serum creatinine (Cr), blood urea nitrogen (BUN), and tissue apoptotic cell levels in the ischemia/reperfusion (I/R) injury-induced AKI rat model following different treatments. Reproduced with permission [88]. Copyright 2019 Elsevier Ltd. **B** (i) the schematic of preparation of chitosan-bilirubin NPs and their mechanism of tumor treatment; (ii) the tumor volume and tumor weight in the HeLa tumor-bearing mouse model after treatment. Reproduced with permission. [90]. Copyright 2020 Elsevier Ltd.** C** Schematic of combination antitumor therapy with ROS-responsive nanogel prepared by in situ gelation of chitosan-thioketal-ferulic acid (CS-FT) conjugate, antiangiogenic Pazopanib (PAZ) and Laccase; (ii) A schematic representation of the synthesis, structure, and preparation of ROS. Reproduced with permission [92]. 2022 KeAi Communications Co. Ltd. **D** The assembly process and treatment mechanism of HA-coated PEI-ss-HECS-ss-OA NPs. Reproduced with permission. Reproduced with permission [84] Copyright 2020 American Chemical Society

Liu et al. prepared pH-responsive, kidney-targeted NPs (size: ~ 50 nm, surface charge: − 19.6 mV) electrostatically complexed with anionic HA (CD44 receptor targeting), cationic chitosan, and antioxidant cationic peptide SS-31 (mitochondria targeting) for the treatment of AKI (Fig. 3A–i) [88]. As the apparent charge of chitosan was susceptible to the low pH condition of lysosomes, the electrostatic equilibrium disruption between chitosan and HA facilitated the precise release of payloads in injured kidney cells. The *i.v.* administration of the formulation in an ischemia reperfusion injury-induced AKI rat model significantly restored renal function (reduced serum creatinine and blood urea nitrogen levels) and possessed excellent mitochondrial protection, oxidation reduction, and inflammation alleviation effects, providing a potential therapy for AKI in future clinical applications (Fig. 3A–ii).

(2) ROS-sensitive chitosan NPs

The modification of chitosan with ROS-responsive functional groups, such as thioketals (TK), thioethers, diselenide bonds, and boronic acid, could accelerate drug release at lesions with high levels of ROS, achieve potent therapeutic efficacy, and reduce off-target toxicity [89–91].

For example, Chen et al. developed a ROS-responsive micelle formed by the self-assembly of chitosan-bilirubin conjugate and evofosfamide (TH-302, a type of hypoxia-activated prodrugs), named TH-302@BR-Chitosan NPs (average diameter is around 100 nm) (Fig. 3B–i) [90]. The hydrophilic chitosan backbone provided a strong intermolecular hydrogen bond between NPs and payload to consolidate drug encapsulation, whereas the grafted hydrophobic bilirubin converted into biliverdin within the ROS-rich tumor region with improved water solubility of the amphiphilic conjugate, resulting in the disassembly of vehicles for releasing the payload [89]. Because TH-302 was activated by BR-mediated deoxygenation, *i.v.* administration of TH-302@BR-Chitosan NPs in the HeLa tumor-bearing mice model displayed significant inhibitory effects with a gradual decrease in tumor growth via hypoxia-activated chemotherapy, as indicated by induced tumor volume and weight, with no recurrence within 19 days, demonstrating excellent anticancer effects (Fig. 3B–ii).

In another study, Chen et al. reported the synthesis of ferulic acid and ROS-responsive TK conjugated chitosan (CS-FT) capable of covalently binding with the antiangiogenic Pazopanib (PAZ) (Fig. 3Ci–ii) [92]. With the injection of CS-FT, hypoxia-activated prodrugs, and Laccase, ferulic acid moieties could react with oxygen via an enzyme (Laccase)-mediated chemical reaction, followed by dimerization, to achieve in situ gelation (CS-FTP gel), while simultaneously consuming oxygen continuously for tumor inhibition. The broken TK bond promoted sustained release of PAZ after exposure to high ROS in the tumor site, promoting drug accumulation in the tumor site. In the 4T1 mouse breast tumor model, the CS-FTP gels treated group delayed tumor growth, inhibited tumor angiogenesis, and extended survival time (40% of mice survived at least 60 days), showing great potential in tumor therapy.

(3) GSH-responsive chitosan NPs

Based on the higher intracellular GSH level (0.5 ~ 10.0 mM) in injured cells than extracellular space (approximately 2.0 ~ 5.0 μM), numerous chitosan-based NPs are used in biomedical applications, particularly immunotherapy and gene therapy, to deliver a variety of biologics to the cytosol specifically in order to achieve desired efficacy [93, 94].

An innovative chitosan amphiphile (PEI-ss-HECS-ss-OA) was developed by incorporating hydrophobic octylamine and cationic PEI with hydroxyethyl chitosan via GSH-responsive disulfide linkages (Fig. 3D) [84]. For the co-delivery of hydrophobic drugs and siRNA therapeutics to the tumor cytoplasm, specifically, the PEI-ss-HECS-ss-OA amphiphile spontaneously assembled to form micelles. After surface coating with HA, the as-prepared micelles showed increased tumor accumulation and cellular internalization, as expected. After hyaluronidase-mediated degradation of the HA coating, the core could achieve GSH-triggered amphiphilic disassembly for co-burst release of the encapsulated two drugs into the GSH-rich tumor cytoplasm, resulting in a remarkable tumor inhibition ratio of 86.6% in A549 lung cancer-bearing mice.

(4) Enzyme-triggered chitosan carriers

It must be noted that lysozyme might degrade chitosan by hydrolyzing the link between N-acetylglucosamine and glucosamine. Especially, the hydrolysis rates are related to the degree of N-acetylation of chitosan [95]. Thus, functionalized chitosan materials with varying degrees of N-acetylation could be employed for the on-demand release of drugs via lysozyme-mediated degradation and applied to diseases with a high lysozyme level (such as wound infection). Furthermore, chitosan could be covalently or non-covalently linked to other enzyme-responsive moieties that selectivity in responding to the over-expressed enzyme (e.g., protease, glycosidase, matrix metalloproteinases, or hyaluronidase, etc.) to cascaded release the entrapped drug at the targeted site. However, covalently synthesized enzyme-triggered drug release biocarriers have a tendency to reduce the specificity of enzymatic processes, affecting the timing of drug release during disease therapy.

In summary, stimuli-responsive chitosan-based smart vehicles have been employed to target a wide range of pathologies by controlling the rate or location of drug delivery in vivo in response to specific stimuli, reducing off-target effects and side effects, which would be advantageous in a variety of clinical settings. 

#### The mucoadhesive chitosan-based carriers for nasal drug delivery

Nasal drug delivery is another route of drug administration that is painless, non-invasive, and does not require sterile preparation. However, cilia-driven mucociliary clearance quickly removes foreign liquid and powder formulations trapped in mucus, resulting in a short half-life (10 ~ 20 min) [96]. The excellent mucoadhesiveness of chitosan, suppression of mucociliary differentiation, and promotion of TJs-opening permeability ensure prolonged interaction between the nanosystems and nasal mucous layer surface and transport of bioactive molecules via epithelial cells (Fig. 1C) [97, 98].

1. Inhalable chitosan NPs for pulmonary delivery

**Fig. 4:**
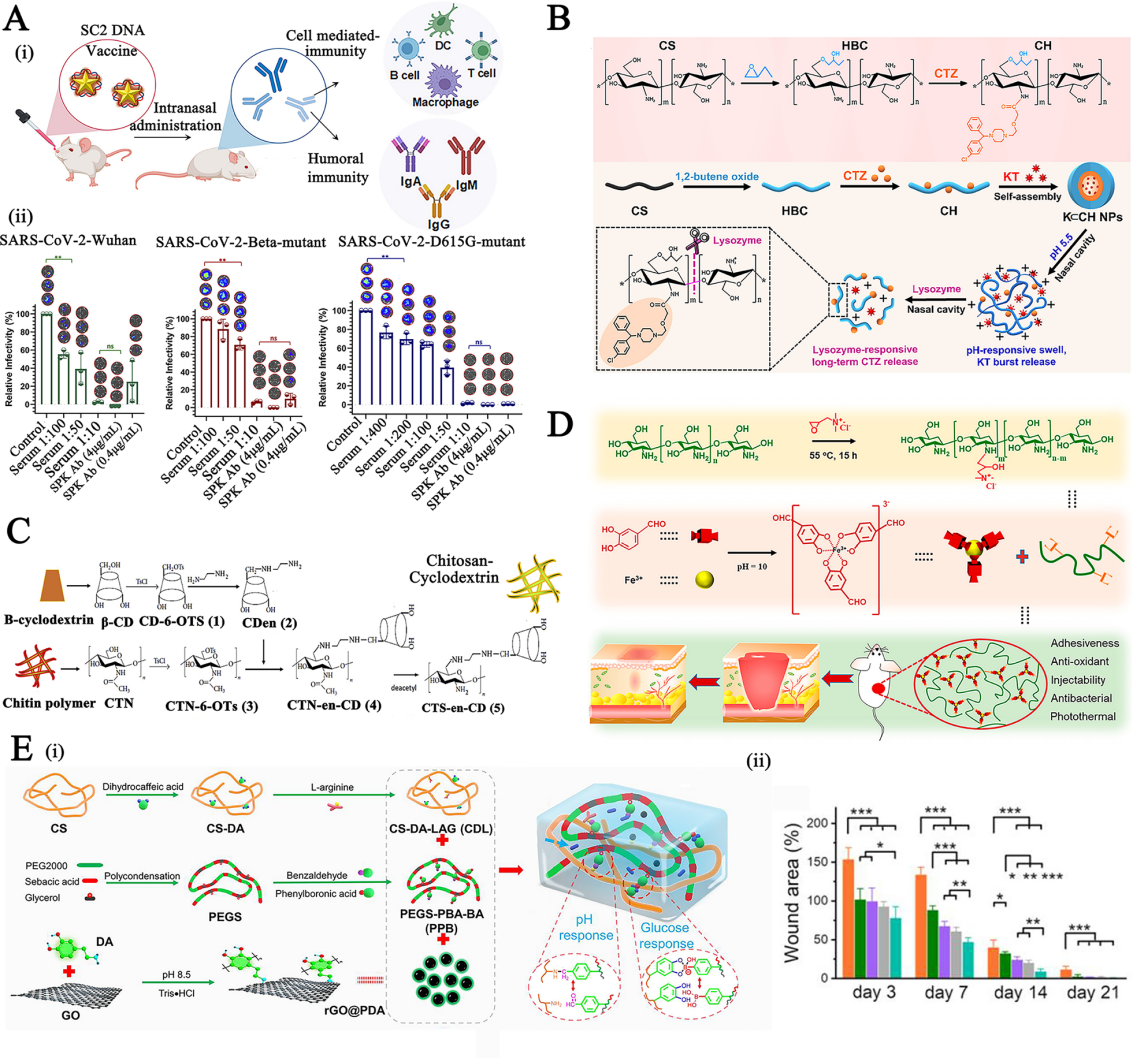
**A** (i) The treatment mechanism of the SC2 DNA chitosan vaccine; and (ii) The relative inhibition of infectivity of the formulation against different lentiviral particles. Reproduced with permission [103]. Copyright 2021 American Chemical Society **B** The preparation and stimulus-responsive drug release mechanism of ketotifen-loaded, cetirizine-modified hydroxybutyl chitosan NPs. Reproduced with permission [104] Copyright 2021 Elsevier B.V. **C** The preparation of β-cyclodextrin-chitosan (CD-CS) hybrid polyfunctional gold-iron oxide NPs. Reproduced with permission. [106]. Copyright 2019 Elsevier Ltd. **D** Illustration of dual-dynamic-bond cross-linked antibacterial adhesive hydrogel composed of ferric iron (Fe^3+^), protocatechualdehyde (PA), and quaternized chitosan (QCS). Reproduced with permission [113]. Copyright 2021 American Chemical Society. **E** (i) Schematic diagram of the preparation and application of metformin-loaded hydrogel; (ii) The wound area evaluation in diabetic foot wound mice model within 21 days. Reproduced with permission [114] Copyright 2022 American Chemical Society

Chitosan vehicles have been considered ideal candidates for muco-inhalable delivery systems for the global coronavirus disease 2019 (COVID-19) pandemic therapeutic [99–101]. Notably, chitosan have strong binding affinity for the SARS-CoV-2 S protein trimer cavity for possessing anti-viral effects and exerting anti-inflammatory effects where lung cellular injury has occurred [102]. For example, Kumar et al. developed a SARS-CoV-2 spike DNA vaccine transported on a gold NPs capping with cationic chitosan polymer (~ 40 nm, + 3.4 mV) via electrostatic complexation for generating a strong and persistent antibody response and neutralizing pseudoviruses expressing S proteins of SARS-CoV-2 variants (Fig. 4A–i) [103]. In addition to its inherent advantages for providing muco-adhesive effects toward respiratory mucosa, the chitosan used here acts as an immuno-potentiating agent to enhance the immunogenicity and efficacy of the vaccine. After nasal administration, the formulation produced high levels of anti-SARS-CoV-2 IgA in the lung mucosa and tissue-resident memory T cells, which nearly completely inhibited the infectivity of different lentiviral particles, highlighting the capabilities of this inhalable SC2 DNA vaccine for inducing a strong mucosal immune response and offering long-lasting protection (Fig. 4A–ii).

Besides, a nasal drug delivery system was prepared by assembling a cetirizine-modified hydroxybutyl chitosan conjugate to encapsulate ketotifen (a histamine inhibitor), yielding NPs for long-term respiratory allergy intervention (Fig. 4B) [104]. Protonation of amino groups in chitosan caused swelling of NPs in the slightly acidic nasal cavity (pH 5.5), and lysozyme-mediated enzymatic (degradation of β-(1–4) glycosidic bonds) hydrolysis of chitosan endowed nasal adaptive sequential drug release with pH-responsive burst release of ketotifen (~ 50% within 12 h) and sustained cetirizine drug release (~ 36% within 72 h), respectively.

(2) Inhalable chitosan nanoparticles for central nervous system administration

Nasal drug delivery of chitosan-based NPs to the central nervous system (CNS) through passive transport across the nasal epithelium as well as the endocytosis pathway of drugs into the olfactory and trigeminal nerves followed by transport by axon terminals was another significant area of intranasal administration [105].

It has been reported that FDA-approved β-cyclodextrin-chitosan (CD-CS) hybrid polymeric NPs can be used for intranasal delivery of cytokines and genetic drugs [106–108]. For example, theranostic polyfunctional gold-iron oxide NPs (which enable MR imaging) were surface functionalized with CD-CS for the loading of miRNAs (anti-miR-21 and miR-100) via electrostatic interactions and then coated with glioblastoma-targeting T7 peptide, yielding NPs with a limiting size below 50 nm (Fig. 4C) [106]. After intranasal delivery in an orthotopic glioblastoma xenograft model, the formulation exhibited progressive accumulation in the brain and was retained for 30 days, resulting in a prominent suppression in glioblastoma proliferation and an increase in mouse survival. This nanoformulation enables multimodal imaging and efficient drug delivery to intracranial tumors, which is favorable for difficult-to-treat forms of cerebral malignancy or other diseases.

Overall, chitosan-based NPs are more likely to reach the respiratory mucosa in the airways and lungs after inhalation, which has significant implications for the treatment of lung-related pandemics [103].

#### Chitosan-based nanomaterials for topical administration

In clinical applications, the treatment of infected skin wounds, postoperative wound management, and post-wound-closure care remain the greatest obstacles. Benefiting from their unique antibacterial properties, adhesion and retention effects at the wound site, and high biocompatibility, cationic chitosan and its derivatives are well-suited for incorporating into antibacterial dressings for skin tissue regeneration or wound healing [101, 109–112].

On the basis of Schiff base bonding and pH-sensitive catechol-metal ion coordination chemistry, Fe^3+^, quaternized chitosan, and catechol- and aldehyde-containing protocatechualdehyde (PA) were used to produce a dual-dynamic-bond cross-linked antibacterial adhesive hydrogel, with PA serving as a motif for matrix bonding (Fig. 4D) [113]. The inherent antibacterial and mucoadhesive capabilities of chitosan and PA’s ability to scavenge free radicals contributed to the hydrogel’s enhanced antibacterial activity, multifunctional adhesiveness, and hemostasis function. Besides, catechol-metal ion coordination possesses a pH-dependent alternation from a weak monocomplex formation at low pH values to a bis- or tris-complex at higher pH values, equipping the hydrogel with on-demand dissolution or removal characteristics. After topical administration, the resulting hydrogel-treated group showed superb blood loss inhibition and promoted healing efficiency in methicillin-resistant *Staphylococcus aureus* (MRSA)-infected full-thickness skin wound mice models and mouse-tail amputation model and the hemorrhaging liver mouse model, allowing it to efficiently close the skin incision.

Liang et al. also synthesized two polymers: cografted chitosan with l-arginine and dihydrocaffeic acid (LD-chitosan) and phenylboronic acid and benzaldehyde bifunctional polyethylene glycol-co-poly (glycerol sebacic acid) (PEGS-PBA-BA). The double-dynamic bonds of a Schiff base and phenylboronate ester bonds between LD-chitosan and PEGS-PBA-BA enabled a pH/glucose dual-responsive release of metformin-loaded hydrogel (Fig. 4E–i) [114]. The mechanical properties, adhesion properties, antibacterial efficiency, hemostatic effect (blood coagulation index), and antioxidant efficiency of hydrogels were significantly improved with increasing LD-chitosan content, providing encouraging results in the healing of diabetic foot wounds in athletes (Fig. 4E–ii).

In addition to their utility in tissue engineering, chitosan-based NPs or hydrogels could also be administered in situ to treat tumors [115–117]. Su et al. prepared injectable peritumoral hydrogels composed of anionic proteins, chitosan, and Ag_3_AuS_2_ nanoparticles via imide covalent and electrostatic interactions for near-infrared (NIR) light-responsive photothermal therapy in tongue tumors [118]. By precisely delivering anti-tumor drugs around the in situ tongue tumor site, this effort has the potential to inhibit tongue tumors and prevent complications. Encouragingly, a pilot phase I/II clinical trial in humans has been executed to improve efficacy and reduce toxicity in patients with resectable oral cavity squamous cell carcinoma by neoadjuvant local administration of a self-adhesive cisplatin transmucosal system (cisplatin-loaded chitosan particles) which undoubtedly providing greater theoretical and practical support for the application of chitosan-based NPs in in situ tumor treatment [116].

#### Commercial development based on chitosan biomaterials

Globally, novel engineered chitosan-based formulations and composites are continuously being developed and have flooded the markets. The majority of chitosan-based biomedical products are Class I devices that are exempt from clinical trials since they are only reassemblies of existing devices or are low risk. Indeed, topical chitosan dressings that do not contain any drugs are excluded from FDA registration and are only required to register and list their products prior to commercialization. A selection of chitosan-based commercial products (including some chitosan-containing hemostatic biomaterials or wound dressings) is included in Table 3. In addition to these, there are still other chitosan-based biomedical products on the market. For instance, KiOmed Pharma has developed an injectable chitosan-based microbead hydrogel (namely Kiome Inevs One) for the treatment of osteoarthritis that has conformite europeenne (CE) approval. Biosyntech’s BST-CarGel^®^ was a commercially available, easy-to-use chitosan-glycerophosphate hydrogel product that could be utilized to promote cartilage regeneration. In a randomized clinical trial, the clinical benefits of BST-CarGel^®^ treatment led to a significant improvement at 12 months post-operatively in patients undergoing cartilage repair. In addition, there are also several innovative ‘‘smart’’ chitosan wound dressings on the market that are capable of stimuli-sensitive state transition. For example, temperature-sensitive hydroxybutyl chitosan gel dressing (Horizon International Medical Deiverce Co., Ltd., China) can undergo gel-sol transformation without affecting the products’s stability. This kind of smart gel has been applied in pyoderma gangrenous and nasal endoscopic surgery with beneficial adjuvant therapeutic effects. The size of the global chitosan market is expected to expand rapidly.

**Table 3:** List of selected chitosan-based or alginate-based marketed biomedical products (mainly hemostatic biomaterials or wound dressing)

Product name	Materials	Functions/Features	Manufacturer	Ref
HemCon^®^	Freeze-dried chitosan acetate salt	Achieving hemostasis in emergency situations	HemCon, USA	[259]
ChitoSAM^TM^100	Chitosan	Hemostatic dressings optimized to stop bleeding fast	SAM^®^ Medical, USA	[260]
ChitoFlex^®^ PRO	Chitosan	External, temporary control of severely bleeding wounds; Providing an external barrier against bacteria	Tricol Biomedical, Inc. USA	[261, 262]
Chitoseal^®^	Chitosan dressing with cellulose coating	For major wounds with bleeding	Abbott, UK	[263]
Clo-Sur P.A.D^®^	Chitosan	Non-woven topical pressure dressing to accelerate hemostasis	Scion BioMedical, USA	[264]
Celox	Chitosan	For the control of massive traumatic bleeding	Sam Medical Products	[265]
TraumaStat^®^	Chitosan, mesoporous silicon and polyethylene	Both hemostatic and absorbent properties High surface area could rapidly interact with the clotting components of blood	Ore-Medix, USA	[266]
Syvek-Patch^®^	High molecular-weight chitin	Claimed to be 7-folds efficient in hemostasis than fibrin glue	Marine Polymer Technologies, Burlington	[263]
Tegasorb^®^	Chitosan-based hydrocolloid dressing	Promoting wound healing; Suitable for leg ulcers, sacral wounds, chronic wounds	3 M Healthcare, USA	[267]
Chitopack C	Cotton-like chitosan	Rebuilding subcutaneous tissues, and regenerating skin regularly	Eisai, Japan	[268]
Algicell^™^	Silver Calcium Alginate	Helping to strengthen dressing when wet; Minimized fibrous residue; Maintaining wound moist environments	Derma Sciences, USA	[269]
Guardix-SG^®^	Alginate and poloxamer	For the purpose of adhesion prevention;	Genewel, South Korea	[270]
Tromboguard^®^	Sodium alginate/calcium alginate, chitosan, silver salt	Immediately stop bleeding; Possessing antibacterial activity for preventing infection	Matopat, Poland	[271]
Pharma-Algi^®^	Alginate, polyurethane,	Stop bleeding; Absorbing the exudate; Keeping the wound moist	Pharmaplast, Egypt	[272]
Alginate	Alginate	Strong absorption capacity; Forming gels when connect with wound exudate; Providing moist environment for wound healing	Winner, China	[273]
Tegaderm^™^	Calcium alginate	Promoting wound healing;	3 M Healthcare, USA	[274]
Alginate wound dressing	Alginate	Efficient absorption of exudate; Promoting wound healing; Pain relief for patients	Shinva Ande Healthcare Apparatus, China	[273]

Notably, while there are currently many chitosan-based dressings on the market, there are no commercially available chitosan-based drug delivery biomaterials. Despite the fact that chitosan’s physicochemical properties (e.g., Mw, DD, etc.) are directly related to its biosafety, few research has been conducted to investigate the metabolic process when these properties are standardized. Few studies have completely addressed the relationship between single or multiple changes in chitosan’s physicochemical properties and their in vivo digestion, absorption, and metabolism, or compared one factor to many studies.

#### Remaining issues and perspectives of chitosan-based nanomaterials

Designing chitosan-based formulations presents a number of significant obstacles that must be addressed, with particular emphasis on the following issues:

1. The safety of orally administering chitosan-based NPs is a primary concern for an approved pharmaceutical formulation. The in vivo biosafety of chitosan as a standalone substance has been proven following oral delivery [119]. However, it should be noted that the presence of various chemical modifications and composite formulations in chitosan NPs does not necessarily guarantee a high degree of biological safety. The occurrence of oscillations in drug concentrations is prone to occur following drug absorption in the digestive system, which can result in a higher incidence of the drug’s adverse effects or inadequate therapeutic outcomes. There is currently a lack of available information regarding the toxicological properties of chitosan-based NPs in clinical trials, particularly in relation to their immune response upon administration orally. Thus, it is imperative to conduct further advanced preclinical studies, including ex vivo investigations, comprehensive long-term toxicity assessments in vivo, experiments with large animals, and retrospective cohort studies, to thoroughly examine the effects of orally administering chitosan-based NPs.
2. In regards to chitosan nanomaterials that respond to stimuli, there is a need for more effective biophysical mechanisms and material design concepts to achieve a potentially reversible conversion of chitosan’s particular morphology instead of the current simple and irreversible disassembly of chitosan vectors in response to biological stimuli. Another field of research that is highly captivating is the development of chitosan vehicles with cascade-reactive drug release. However, it is important to note that these investigations are currently constrained in their scope. Furthermore, it is suggested that future research prioritize examining the correlation between the responsiveness of chitosan NPs and their physicochemical properties. This will enable the development of nanomaterials with the ability to accurately detect and quantify stimulus concentrations. This approach will harness the most relevant stimuli in vivo to achieve quantifiable drug release that remains unaffected by variations in other factors, resulting in the personalization of treatment regimens for individual patients.
3. In addition to live-attenuated viral vaccines, the application of inhalable chitosan for the development of nano-vaccine formulations with high biocompatibility and immunoregulatory function has emerged as a promising alternative strategy. This approach holds the potential to significantly reduce immunogenicity and minimize side effects, thereby offering considerable benefits to patients, particularly those with compromised immune systems [120]. Nevertheless, additional investigation into the correlation between the physicochemical properties of chitosan and their immunoactivation effects was not pursued. Previous studies have noted that chitosan with complete deacetylation (100% DD) has optimal efficacy as a vaccine adjuvant, boosting antibody responses and facilitating the development of cell-mediated immunity. However, this advantage may be counterbalanced by reduced water solubility and accelerated rates of biodegradation [101]. The enhancement of in vivo therapeutic efficacy of carriers in immunotherapy can be achieved through the optimization of chitosan DD, deacetylation pattern, and molecular weight.
4. In addition, it should be noted that residual allergenic contaminants of chitosan during the classic chemical purification process or biosynthesis procedures may cause potential adverse effects [121]. For instance, the leftover proteins in chitosan, such as tropomyosin and arginine kinase from shrimp shells, can cause severe inflammation, allergic reactions, and immune rejection in living organism [122, 123]. Consequently, the amount of residual protein in chitosan is crucial in determining its suitability as a medical material. The National Medical Products Administration of China published the pharmaceutical industry’s “Chitosan in Tissue Engineered Medical Devices” (YY/T 1699–2020) guideline, which states that the amount of leftover protein in medical-grade chitosan should not exceed 0.2 wt%. Numerous studies have extensively investigated the enhancement of chitosan's deproteinization ability and reduction of protein residues through improved enzymatic degradation and biological fermentation. However, this process typically requires expensive apparatus, and the use of acids and alkalis is still required after deproteinization. Specifically, it can be difficult to achieve precision control over the chemical structure and molecular weight of chitosan during the enhanced purification process. Therefore, when employing chitosan in widespread biomedical applications, it is essential to address the purification of source materials in order to minimise immunogenicity and mitigate potential adverse effects.

### Chondroitin sulfate-based nanomaterials

Chondroitin sulfate (CS), a glycosaminoglycan, is made up of disaccharide units (i.e., N-acetyl-β-d-galactosamine and β-d-glucuronic acid residues) with sulfate groups at different locations. In comparison to CS extracted from terrestrial sources, which contain non-sulfated or monosulfated units, CS produced from marine organisms (generally extracted and purified from cartilaginous fishes, particularly sharks) contains a greater proportion of disulfate units. This structural differentiation confers specific biological activities, such as antioxidation, anti-inflammatory, antitumor, and immune-regulatory activity, among others. Besides, the sulfate group-induced formation of an external charge barrier and the higher hydrodynamic volume of CS during circulating may inhibit undesirable interactions with plasma proteins and cells, hence improving colloidal stability [124–127]. The efforts in the design of CS-based functional drug delivery systems were divided into three major categories:

1. CS-based NPs for precise drug delivery to specific cells

Recent research has modified CS with hydrophobic segments or coupled it with other cationic polymers to form nanosystems for effective tumor targeting by the strong interaction of CS with CD44 receptors [19, 101, 128–130].

**Fig. 5:**
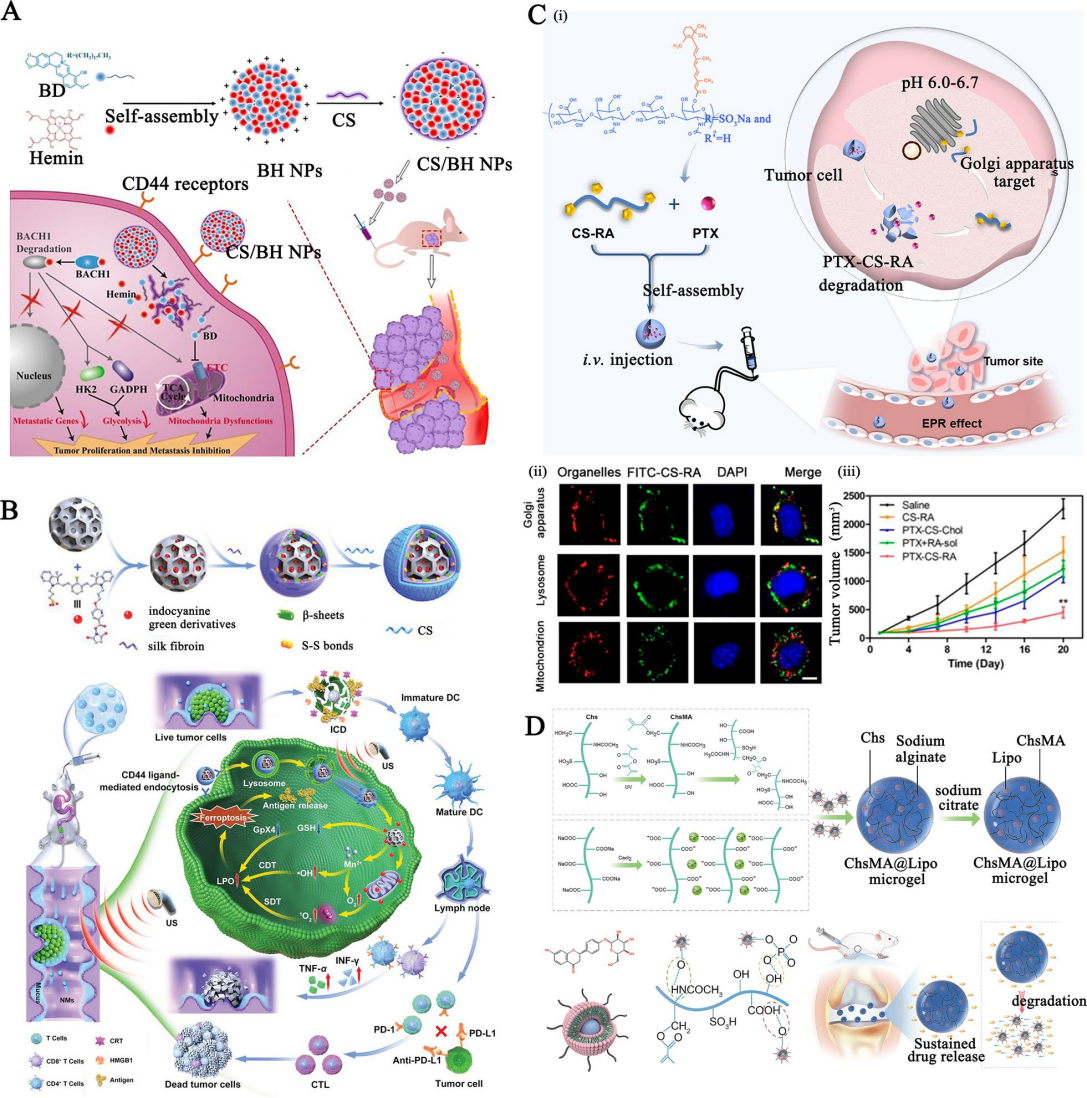
**A** Schematic of chondroitin sulfate (CS)-coated berberine derivative (BD)/hemin self-assembled nanoparticles (CS/BH NPs) and its anti-tumor mechanism. Reproduced with permission. [117]. Copyright 2023 Elsevier B.V. **B** The preparation and treatment mechanisms of mesoporous manganese oxide (MnOx)-based nanomotors that surface coated with silk fibroin and CS. Reproduced with permission [76]. Copyright 2022 John Wiley & Sons, Inc. **C** (i) The schematic illustration of the assembly of CS-retinoic acid and anti-tumor drug paclitaxel (PTX); (ii) Location of NPs in different organelles; (iii) The tumor volume of 4T1-bearing mouse models treated with different formulations. Reproduced with permission. [133]. Copyright 2019 American Chemical Society **D** The preparation of liquiritin-loaded gel utilizing photo-cross-linkable methacryloyl group-modified CS. Reproduced with permission. [135]. Copyright 2022 Acta Materialia Inc

For example, Yang et al. fabricated BH NPs formed by BACH1 (transcription factor BTB and CNC homology 1) inhibitor hemin and mitochondria function inhibitor berberine derivative (BD) via nano-precipitation method followed by surface ccoating of CS via strong electrostatic interactions, yielding tumor targeting CS/BH NPs (size: 141.3 nm, surface charge: − 23.1 mV) (Fig. 5A) [117]. When administered intravenously to MDA-MB-231 tumor-bearing BALB/c nude mice, CS-mediated CD44 targeting enhanced tumor accumulation by 1.9-fold at 8 h and prolonged retention at the tumor region by up to 24 h, compared to that of the free drug group, thereby laying the groundwork for subsequent tumor inhibition, resulting in remarkably decreasing tumor cell volume and reduced expression of metastasis-associated proteins, suggesting potential clinical significance for the treatment of malignancy.

Besides, CS could be prepared for oral drug delivery systems targeting CD44-overexpressed colonic macrophages via receptor-mediated endocytosis [131]. For example, curcumin was encapsulated into the hydrophobic region of amphiphilic silk fibroin (with pH- and GSH-responsiveness) to yield multiple-responsive carriers with micellar structures, which were subsequently coated with CS for macrophage targeting. In another study, an indocyanine green derivatives-loaded mesoporous MnO_*x*_ was surface coated with CS and silk fibroin followed embedded into the chitosan/alginate hydrogel to attain a multi-functional drug delivery system (Fig. 5B) [132]. After oral administration, this system was specifically internalized by colon tumor cells via CS-mediated CD44 targeting and then released the payloads by the responsive degradation of silk fibroin. The released preferentially gathered around mitochondria and the released Mn^2+^ were synergistically catalyzed endogenous H_2_O_2_ into toxic OH via Fenton-like reactions, achieving dual-targeted treatment of colon tumors by direct synergistic tumor suppression and combined utilization of immune checkpoint inhibitors.

(2) CS-based NPs for precise drug delivery to specific organelles

CS may also interact with N-acetylgalactosaminyltransferases when targeting the Golgi apparatus, which comprises a collection of glycosylation enzymes that attach sugar monomers to proteins as they travel through the apparatus [115, 133]. As proof of concept, Gong et al. prepared Golgi apparatus-targeting NPs (size: 182 nm, surface charge: − 18.6 mV) composed of amphiphilic retinoic acid (RA)-conjugated CS (CS-RA) and anti-tumor PTX (Fig. 5C–i) [133, 134]. The hydrophilic main chains CS primarily deliver cargo to subcellular organelles (Golgi apparatus), where the RA exerts anti-tumor effects by facilitating structural damage to Golgi and interfered tumor metastasis-associated protein expression (Fig. 5C–ii). In a mouse model of hepatocellular carcinoma, this formulation possessed promising therapeutic potential by shrinking the primary tumor mass, inhibiting tumor metastasis, prolonging survival time, and causing minimal to no side effects (Fig. 5C–iii). This strategy achieved subcellular targeting in tumor cells and precise delivery in the Golgi, which will assist cancer patients.

(3) CS-based nanogel for drug delivery

Furthermore, because of the large amount of active functional groups, such as COO^−^ and SO_3_^−^, hydrophilic CS could be considered a potential candidate for hydrogel networks capable of absorbing large amounts of water due to its chemically modifiable groups capable of both covalent and noncovalent bonding. Assisting with its chondrogenic phenotype-maintaining capabilities, CS has been developed as an injectable hydrogel for the treatment of osteoarthritis (Fig. 5D) [135]. To be more specific, CS was covalently modified with photo-cross-linkable methacryloyl groups (ChsMA) and then fabricated as ChsMA gel for encapsulating liquiritin-loaded liposomes (LQ, a hydrophobic antioxidation agent), with alginate serving as a sacrificial material. In the inflammatory articular cavity, hyaluronidase may degrade the β-N-acetylhexosamine-1,4 glycosidic bond of the CS moiety, resulting in sustained release of the cargo. The LQ drug combines with the degradation products of CS to eliminate ROS in a synergistic manner, thereby slowing the progression of osteoarthritis.

CS is widely used in drug delivery systems as building blocks or functionalization units to pursue active targeting, improved biocompatibility, high efficiency, versatility, and synergistic therapy with payloads. Nonetheless, the majority of current studies that include CS-based drug delivery systems do not distinguish between the various sources of CS. The high relative abundance of sulfated units in marine CS is particularly noticeable compared to terrestrial CS. Notably, commercially available CS has a mixture of CS disaccharide motifs with varying ratios or varying molecular weights, is derived from a variety of marine species, and contains a number of inert contaminants. Using marine CS with varying amounts of sulfation, future studies may be aimed at investigating the therapeutic effects and particular biological properties of CS.

### Alginate-based nanogels

Alginate is derived mostly from the cell walls of algae and consists of (1–4)-linked β-mannuronic acid (M-block) and α-guluronic acid (G-block) residues. The content and sequence of G/M residues vary based on the types of sources and species employed to extract the compounds. Interactions between the carboxyl and hydroxyl groups of G- blocks and cations (Ca^2+^, Mg^2+^) have been reported as a means of producing hydrogels (ionotropic gelation) [136]. Generally, alginates with a high G-block content generate hydrogels with higher mechanical strength, whereas alginates with a high M-block concentration are more likely to form softer, more elastic hydrogels. However, several studies have demonstrated that alginate with a high M-block content exhibits higher immunogenicity. Besides, a rise in the Mw of alginate polymer chain would enhance its viscosity. The high Mw of the alginate would hamper the clearance rate of alginate chains in vivo. Consequently, the chemical structure of alginate must be fully considered during the preparation of gels.

#### Alginate nanocomposites for drug delivery or tissue engineering

1. Physically noncovalent cross-linking alginate hydrogels

The alginate could be gelled using divalent cations as the cross-linking agent under mild conditions, and the resultant hydrogel exhibited high water content, elasticity, permeability, and the capacity to contain a moist environment; the hydrogel has been widely used in wound healing and tissue regeneration owing to its similarity to ECM environments [42, 130, 137–144].

**Fig. 6:**
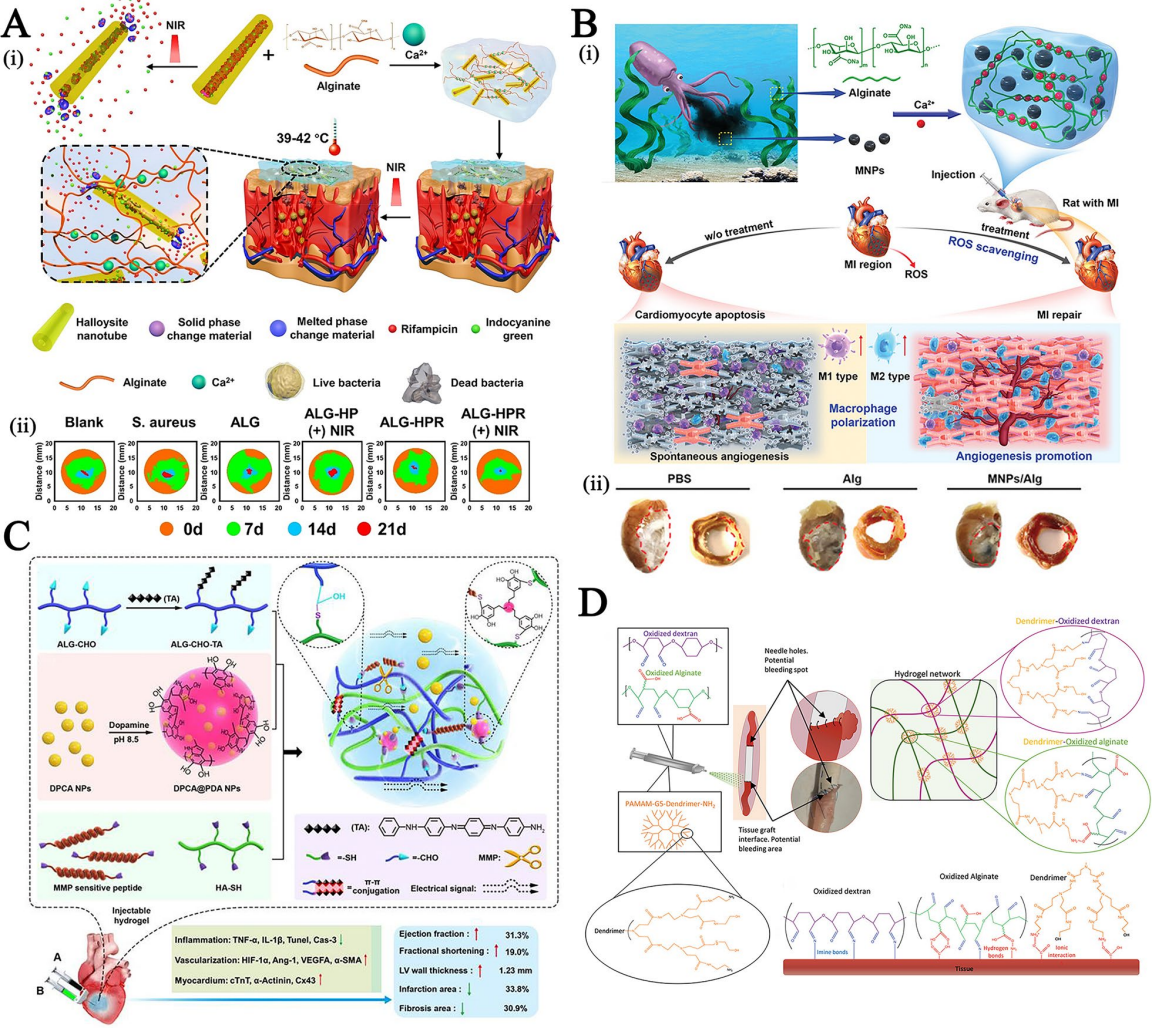
**A** (i) The scheme for the preparation of phase-change material (a eutectic mixture of fatty acids)-gated nanotubes that were integrated into alginate hydrogel and the NIR-triggered release of the payloads; (ii) The traces of wound closure in an infected full-thickness skin wound model in rats after 21 days of treatment with different formulations. Reproduced with permission. [145]. Copyright 2022 Elsevier B.V. **B** (i) A schematic of melanin NPs/alginate hydrogel preparation and therapeutic mechanisms in cardiac repair; (ii) The effects of MNPs/Alg hydrogels on the morphology of infarcted hearts after 28 days. Reproduced with permission. Copyright [149]. Wiley‐VCH GmbH** C** Preparation of an MMP-sensitive hydrogel (NMP-SP) consisting of partially oxidized alginate and a 1,4-dihydrophenonthrolin-4-one-3-carboxylic acid (DPCA) nanodrug decorated with polymerized dopamine (DPA) and cross-linked with thiolated HA (HA-SH) and thiolated matrix metalloproteinase-sensitive peptide. Reproduced with permission. [152]. 2023 Ivyspring International Publisher **D** Schematic of the biomaterials based on oxidized alginate and oxidized dextran in combination with polyamidoamine (PAMAM) dendrimer amine and the interaction of each polymer with biological tissue. Reproduced with permission. [153] 2022 Wiley–VCH GmbH

For example, a smart hydrogel was prepared by integrating halloysite nanotubes (co-encapsulating the antibacterial drug rifampicin, a NIR-absorbing dye, and phase-change materials (a eutectic mixture of fatty acids)) into alginate at 25 wt% and cross-linking it with divalent cations (Ca^2+^), allowing *in-situ* gelation and preserving the integrity of drug-loaded nanotubes (Fig. 6A–i) [145]. The alginate provided a structural backbone with enhanced mechanical stability for the entire formulation, enabling either the enhancement of the therapeutic effects of the payload against bacteria or the promotion of wound healing. The resultant formulation exhibited good antibacterial activity (continuous bacterial inhibition by 7 days) and accelerated wound healing (completely regenerating the wound and covering it with new tissues at 21 days) in a rat infected full-thickness skin wound model (Fig. 6A–ii).

Alternatively, injectable alginate hydrogels have been designed for cardiac tissue repair after myocardial infarction [146–148]. Zhou et al. proposed functional ROS-scavenging hydrogels (MNPs/Alg hydrogels) for myocardial infarction (MI) treatment using melanin nanoparticles (MNPs) obtained from cuttlefish ink and alginate purified from marine algae via the divalent cations (Ca^2+^) cross‐linking (Fig. 6B–i) [149]. Anionic polysaccharide alginate provides appropriate mechanical support for the heart’s injured area. MNPs may promote macrophage phenotype polarization (from M1 to M2 macrophages), resulting in anti-inflammatory effects. Besides, the plentiful reductive functional groups of MNPs endow the NPs with strong free radical scavenging abilities, minimizing cardiomyocytes oxidative stress injury. MNPs and Alg hydrogels played combined roles in regulating inflammatory MI microenvironments, stimulating angiogenesis, and lowering infarct area in MI mice after 28 days of treatment, significantly improving cardiac function recovery (Fig. 6B–ii).

Besides, in the application of wound treatment, Jiang et al. proposed an innovative bionic hydrogel prepared by covalent amidation crosslinking by the abundant amino groups of natural platelet and active carboxyl groups of alginate [140]. The excellent antibacterial agent, AgNPs, was added to the composite hydrogel and furtherly avoid the abuse of antibiotics. The platelets acted as blood clotting agents that released vasoconstrictors to block the injured vessels and promoted clotting during epidermis damaged. After applied in the mouse model of skin wound infection, the results demonstrated the bio-inspired composited hydrogel promoted hemostasis of acute tissue injury, prevented bacterial proliferation, promoted angiogenesis and granulation tissue formation in wound healing.

In a recent study, Huang et al. developed an in situ assembled trapping gel composed of recombinant glutamate decarboxylase 67 (rGAD67) protein, alginate, and temperature-sensitive Pluronic F-127 for the direct elimination of glutamate (Glu) and free Ca^2+^ released from injured cells during spinal cord injury (SCI) secondary damage [150]. Following intrathecal administration, Pluronic F-127 promoted in situ gelation while free Ca^2+^ auto-exchanged with Na^+^ from sodium alginate to form gellable calcium alginate. Additionally, Glu was captured by rGAD67 and decarboxylated into neuroprotective γ-aminobutyric acid. The elimination of Glu and Ca^2+^ facilitates the recovery of SCI as evidenced by reduced lesion volume (decreased ~ 80%) and inflammatory response (significantly reduced TNF-α, IL-1β, IL-6 level) in a rat model of SCI, providing a unique, minimally invasive synergistic strategy for SCI repair.

(2) Partially oxidized alginate-based nanogels for enhancing the biodegradation

However, due to their high crosslinking density and the lack of degradation enzymes of in vivo, bio-inert, some of the alginate hydrogels are not readily biodegradable in vivo, which may impede cell infiltration and neo-tissue formation during tissue repair and functional integration. Notably, studies have revealed that partially oxidized alginate forms weaker hydrogels in the presence of cations, but this leads to faster degradation, which aids in achieving more prominent biosafety [151]. Furthermore, enzymatically degradable matrix (such as matrix metalloproteinase-sensitive peptides, or MMPs) was used as crosslinkers to build hydrogel networks for rationally controlled degradation or therapeutics delivery. For example, Wei et al. used partially oxidized alginate (ALG-CHO) and 1,4-dihydrophenonthrolin-4-one-3-carboxylic acid (DPCA) nanodrugs decorated with polymerized dopamine (PDA) to cross-link with thiolated HA (HA-SH) and thiolated matrix metalloproteinase-sensitive peptides (MMP-SP) to form MMP-sensitive hydrogel for recovering cardiac functions (Fig. 6C) [152]. Both the partially oxidized alginate and the MMP-sensitive moiety confer increased biodegradability and rationally controlled degradation behavior to the hydrogel networks. The multifunctional hydrogel with good biocompatibility remarkably improved the microenvironment of the infarcted area (holding the slightest collagen deposition and the thickest left ventricular wall thickness) and rescued infarcted heart function. Future research should focus on elucidating in-depth the interactions between the designed injectable hydrogels and cardiac-related cells (e.g., primary cardiomyocytes and fibroblasts).

Besides, Taboada et al. prepared a sprayable, two-component hydrogel composed of G5 polyamidoamine (PAMAM)-dendrimer-amine and oxidized dextran and oxidized alginate, whose aldehyde groups form reversible covalent bonds with tissue amines (Fig. 6D) [153]. The carboxylic groups of oxidized alginates can interact with PAMAM dendrimer both internally and externally via aldehyde-amine interactions, allowing for the creation of a robust material capable of withstanding supraphysiological pressures (≈ 300 mmHg). The imine chemistry-created hydrogels produce reversible bonds that could be hydrolytically degraded over time without the need for additional enzymes, thereby releasing the initial polymeric components. In vitro cell viability and 30 day in vivo subcutaneous implantation biocompatibility studies (in accordance with the FDA guidelines) exhibited the high biosafety of the formulation. The as-prepared hydrogel is capable of preventing bleeding and providing satisfactory sealing of a rabbit aortic puncture and a carotid-graft interface in a pig model.

In addition, as the crosslinking density of alginate hydrogels is proportional to the number of carboxyl groups on polymer chains, functional moieties or therapeutic molecules are grafted onto the alginate backbone to occupy a portion of the carboxyl groups, thereby increasing biodegradability. Deferoxamine (a commercial medicine that could stimulate neovascularization) was grafted into alginate via amidation reaction to form hydrogels, for instance. This kind of grafted hydrogel exhibited a faster degradation rate, superior tissue infiltration, and an accelerated wound healing rate, offering a promising strategy for tissue regeneration [154].

(3) pH-responsive alginate gels for controlled drug release

It was intriguing to note that pH sensitivity is another peculiarity of alginate. Due to a decrease in repulsion between protonated carboxylic groups, the biopolymer of alginate hydrogel tends to shrink at acidic pH, which may restrict payload release from hydrogel networks. However, alginate gels expand in alkaline environments as a result of electrostatic repulsion generated by carboxylic acid dissociation, hence boosting drug release [155, 156]. Utilizing the pH-responsive swelling properties of alginate, a multistage-responsive hydrogel was prepared using a polymeric nanocapsule and alginate through in situ free-radical polymerizations, in which gene drugs were loaded into a nanocomplex [157]. The pH-responsiveness of alginate made it possible for the hydrogel to reach the colon (pH > 7.0) within 2 h and then increased the rate of drug release. As alginates are easily degraded by an enzyme produced by colon bacteria, the payloads were subsequently transported to the colonic lumen, thereby facilitating the accumulation and distribution of gene drugs at the lesion.

On the other hand, alginate/chitosan hydrogel refers to a class of biomaterials that shield payloads from premature degradation throughout the stomach and release drugs sequentially in the intestine, providing a promising research trend in oral delivery (see  ‘‘Chitosan-based nanomaterials for enhanced oral drug delivery’’ section for more details).

(4) Alginate copolymer-based NPs for drug delivery

In addition to alginate hydrogel, the current study has generated an amphiphilic triblock polymer using alginate domains with polylactic acid (PLA) and PEG, which may be assembled into NPs for drug delivery. In contrast to conventional formulations that are restricted to encapsulating one type of hydrophobic or hydrophilic drug, the NPs are capable of encapsulating both hydrophobic (irinotecan) and hydrophilic (azathioprine, DOX) small molecule payloads, broadening their potential as a drug delivery system for combination therapy [158].

#### Alginate-based biomaterials in markets

Numerous alginate dressings have been commercialized and authorized for marketing [127]. Current alginate-based commercial dressings can be divided into surface dressings and wound fillers. Compared to conventional wound dressings, the cross-linked G-chain of alginate can form hydrophilic pores to maintain wet healing environments for wounds while also releasing crosslinking metal ions Ca^2+^ to trigger blood coagulation and accelerate wound healing. Besides, alginate can prevent the invasion of harmful pathogenic microorganisms by forming a physical barrier and activating macrophages at wound sites. Alginate’s hydrophilic nature enhances the absorption of exudate while preventing excessive dehydration of wounds, making it an excellent treatment for severe exuding wounds.

Some research on alginate-based biomaterials has resulted in the initiation of clinical trials for a variety of applications, including the encapsulation of β-cell islets for diabetes therapy (DIABECELL^®^, ClinicalTrials.gov Identifiers NCT00940173, NCT01736228, and NCT01739829), intracoronary delivery of alginate biomaterials in myocardial infarction (MI) treatment (ClinicalTrials.gov Identifiers NCT00557531) and dilated cardiomyopathy (ClinicalTrials.gov Identifier NCT00847964, NCT04781660), mesenchymal cell encapsulation in space-occupying intracerebral hemorrhage therapy (GLP-1 CellBeads^®^, ClinicalTrials.gov Identifier NCT01298830), among others [143, 159]. These ongoing clinical trials undoubtedly provided more definitive answers about the functional benefits of the aforementioned biomaterials in the treatment of various diseases. More efforts must be made in the development of “smart” alginate-based release systems for the sequential and controlled release of multiple drug combinations, thereby improving biosafety, enhancing sustained and local treatment effects, and providing superior therapeutic outcomes. Especially for tissue engineering, the controlled mechanical stability of alginate biomaterials appears to be a critical aspect of the fabrication process in order to form more cell-interactive biomaterials, thereby exerting a great degree of control over cell growth, organization, differentiation, and functions.

### Fucoidan-based nanomaterials

Fucoidan is a sulfated anionic polysaccharide found in the cell wall matrixes of brown seaweed. The backbones of fucoidan consist of α-1,3-linked fucose and α-1,4-linked fucose with substitutions of sulfate groups at the C-2 and C-4 locations, and occasionally at the C-3 positions. Fucoidan is variable in composition, as it may contain heterogeneous monosaccharide branching (xylose, arabinose, rhamnose, glucose, uronic acid, etc.). Notably, fucoidan was approved by the FDA as a GRAS product for use in food additives. Recent research on fucoidan in drug delivery mainly utilizes its three properties:

1. P-selectin targeted fucoidan-based NPs

P-selectin is an inflammatory cell adhesion protein that is upregulated on both endothelial and platelets during cancer, tumor metastasis, endothelial dysfunction, platelet activation, and other conditions.

Specifically, in certain inflammatory diseases, the inflammatory response is regulated by the molecular interaction between functional carbohydrate moieties (such as the O-linked tetrasaccharide Sialyl-LewisX motif of P-selectin glycoprotein ligand-1 (PSGL1) on the surface of leukocytes or sulfated glycosaminoglycan) and the selectin family on the endothelium. Due to its competitive binding to the highly expressed P-selectin on endothelial cells, the algae-sulfated glycan fucoidan could help reduce carbohydrate moieties-mediated leukocyte recruitment on endothelium and attenuate excessive infiltration of leukocytes at inflammatory zones, allowing it to be used as an inflammation regulator and ROS scavenger in pathological tissues. In addition, fucoidan has been reported to inhibit intracellular inflammatory cascades and slow down inflammatory tissue damage by inhibiting the activation of the NF-κB, MAPK, and Akt signal pathways. This also offers potential synergistic therapeutic effects while facilitating the active targeting of fucoidan-based nanomaterials [160, 161]. Fucoidan-based and fucoidan-coated nanomaterials were designed based on the specificity of fucoidan ligands to P-selectin receptors in order to target endogenous and stimulus-induced overexpressed P-selectin [162–165].

**Fig. 7:**
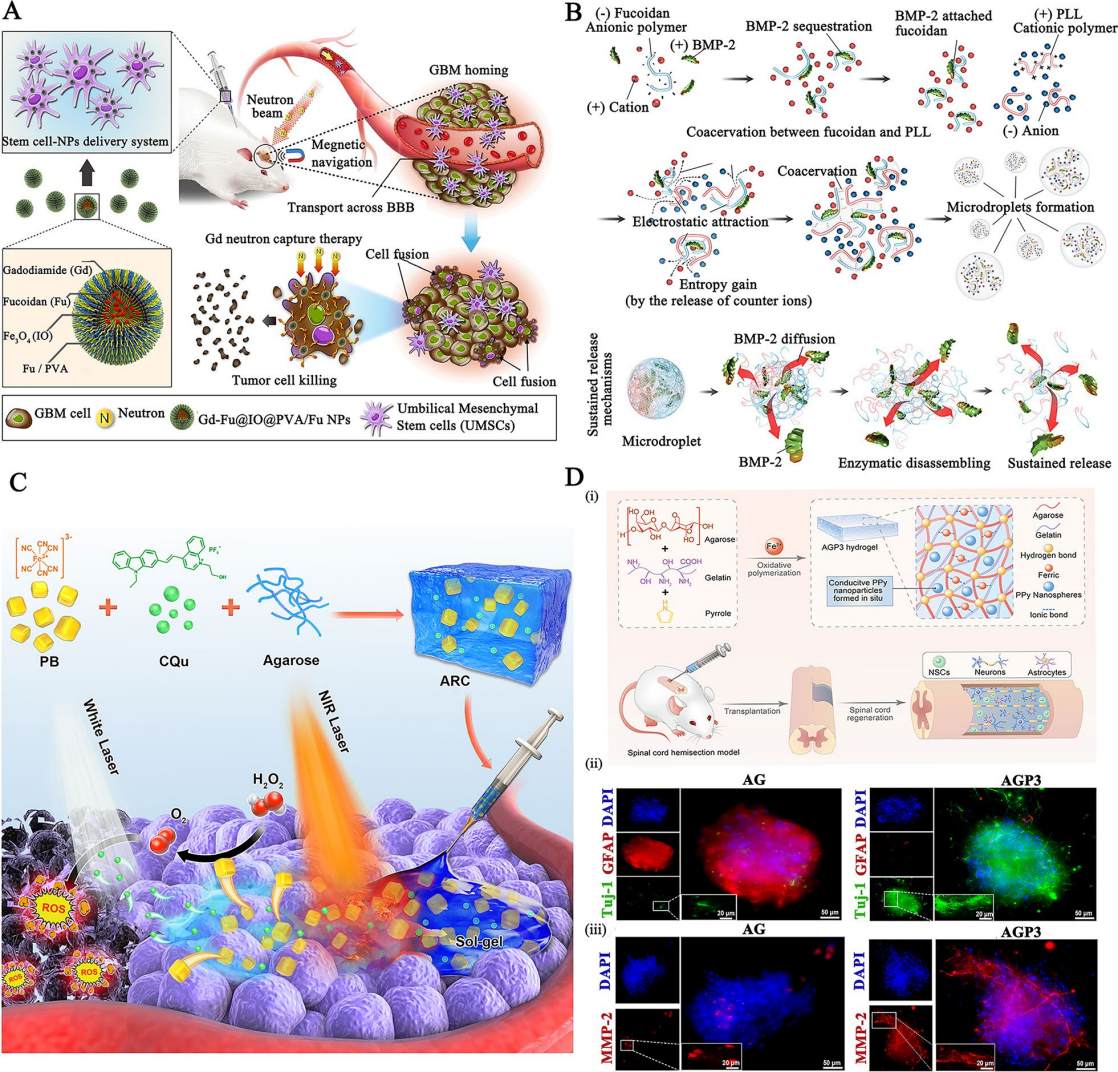
**A** Schematic gadodiamide-fucoidan@PVA/fucoidan NPs associated with umbilical cord mesenchymal stem cells (UMSCs) and the anti-tumor therapy. Reproduced with permission. [166] Copyright 2023 Springer Nature Limited. **B** The formation of BMP-2 encapsulation in fucoidan/PLL complex coacervate system and the sustained protein mechanism. Reproduced with permission [172]. Copyright 2022 John Wiley & Sons, Inc. **C** Schematic illustration of Prussian blue (PB) and aggregation-induced emission luminogen (AIEgen) co-encapsulated agarose hydrogel. Reproduced with permission [183]. Copyright 2021 Elsevier Ltd.** D** (i) Schematic of the preparation of agarose/gelatin/polypyrrole hydrogels; (ii) The expression of Tju-1 (newly generated neurons) and MAP-2 (mature neurons) following different treatments. Reproduced with permission. [184]. Copyright 2021 KeAi Communications Co. Ltd

Lai et al. developed nanoparticle system (Gd-Fu@IO@PVA/Fu NPs) prepared by PVA and fucoidan NPs for Gadodiamide and Fe_3_O_4_ (IO) encapsulation following by surface coating with mesenchymal stem cells derived from umbilical cord (UMSCs) (Fig. 7A) [166]. This formulation was a dual-targeting drug delivery stems that was mediated by stem cell homing and fucoidan targeting for gadolinium-neutron capture therapy (NCT) and real-time MR imaging in glioblastoma multiforme (GBM) management. UMSCs served as a cellular vehicle to penetrate the blood brain barrier (BBB) for achieving the precise delivery of nanoformulation gadodiamide. Furthermore, the fucoidan enhanced tumor cellular association by P-selectin, whereas the formulation gadodiamide shielded by a dense surface composed of fucoidan and PVA exhibited reduced cytotoxicity toward UMSCs during the journey toward homing and cell fusion. Notably, the fucoidan mediated anti-inflammatory and anti-oxidative properties synergistically reduced the oxidative stress injury via Akt pathway and promoted nerve repair after radiotherapy. This bio-inspired delivery systems provided an alternative strategy for the brain diseases treatment.

Fucoidan can also be utilized directly as a surface coating on a variety of formulations for preferentially targeting P-selectin [165, 167–169]. For example, Chung et al. fabricated fucoidan-modified polyamidoamine (PAMAM) dendrimers nanosystems (size: ~ 206 nm, surface charge: − 27 mV) by means of electrostatic interactions for the co-delivery of hydrophobic photosensitizer and MnO_2_ NPs [170]. Owing to the P-selectin targeting effects of fucoidan and the GSH-responsive properties of bioreducible PAMAM, the payloads were precisely released at the breast cancer cells and vasculature after dendrimers disintegrated completely. MnO_2_ stimulated the catalytic generation of O_2_, which alleviated the hypoxia of the tumor spheres, hence rendering photodynamic treatment possible. The administration of the resulting NPs with light irradiation to a mouse model of an orthotopic breast tumor resulted in reduced tumor growth and metastasis.

Notably, a recent study revealed that DOX-loaded fucoidan-deoxycholic acid micelles (with a size of roughly 120 nm and a surface charge of − 20 mV) could hitchhike on activated platelets via the P-selectin receptor [171]. Due to the inherent tumor-homing capabilities of platelets, platelet-hitchhiking micelles would precisely track and identify tumor cells. At various time intervals (1, 8, and 24 h), the accumulation of fucoidan-functionalized micelles in tumors is higher than that of free drug and dextran-functionalized micelles, thereby facilitating the remodeling of the microenvironments of primary tumors and metastatic tissues. After systemic administration in a mouse model of 4T1 spontaneous metastasis, this micelle dramatically reduced tumor volume, induced tissue necrosis, and inhibited proliferation activity. The as-prepared micelle treatment group exhibited robust inhibitory effects on both lung and liver metastasis, with the nodules becoming almost invisible.

(2) Fucoidan-based systems for controlled therapeutic protein delivery

As a kind of glycosaminoglycans, fucoidan also be an attractive candidate for controlled delivery of proteins, as it could specifically bind the domains of proteins via various interactions, i.e., electrostatic, van der Walls interactions, and hydrogen bonding.

Jeon et al. developed a fucoidan-based complex coacervate based on the electrostatic binding of anionic fucoidan, cationic bone morphogenetic protein-2 (BMP-2), and cationic polymer PLL to deliver model protein in a sustained manner for prompting and robust bone regeneration (Fig. 7B) [172]. Fucoidan provided a strong interaction with protein higher than that of heparin (commonly used polymers for proteins binding), hence mediated a reduced burst release within the first day and a prolonged sustained of BMP-2 at the followed tested period (60 days). After applied in the rat calvarial bone defect model, the as-prepared systems promoted instigated localized and targeted bone regeneration in vivo and promoted the quality of newly formed bone indicated increased bone density, which opening possibilities for coacervates to be applied toward controlled release biomaterials for regenerative medicine.

(3) Bacteria-targeting fucoidan-based NPs

In addition to tumor-specific P-selectin-dependent targeting strategies of fucoidan, one study has demonstrated the targeted action of fucoidan against *Helicobacter pylori* (*H. pylori*) via interacting with the several fucoidan-binding proteins on the *H. pylori* surface. Zou et al. constructed fucoidan-coated NPs via simple self-assembly of metformin-linoleic acid and linoleic acid, encapsulating the urase inhibitor ebselen (binding the active site of urase for exerting anti-*H. pylori* activities), and fucoidan was coated on the surface, yielding NPs referred to as FU/ML-LA/EB NPs (particle size: ~ 150 nm, surface charge: − 50 mV) [173]. The negatively charged fucoidan surface coating facilitated NP penetration through the gastric mucus layer and accelerated their deep penetration into *H. pylori* biofilms, increasing drug delivery efficiency. Besides, the fucoidan coating also competitively inhibited *H. pylori* adhesion via blocking the binding of blood group antigen-binding adhesin (BabA) on H. pylori to Lewis b antigens on host cells, hence preventing biofilm regeneration. After oral administration of the formulation to *H. pylori*-infected mice, nearly no residual *H. pylori* was found in the mice, confirming the superb treatment effects of the antibiotic-free strategy.

Even though ongoing research is focused on their multidirectional bioactivities and applications in drug delivery, no fucoidan-based drug products have yet to be registered. Fucoidan is commonly used as a functional food and dietary supplement for patients suffering from a variety of diseases. It is noteworthy that fucoidan alleviated the side effects of platinum-based chemotherapy in the III-IV stage of clinical trials (ClinicalTrials.gov Identifier: NCT03130829). In clinical practice, fucoidan’s benefits in lowering adverse effects and increasing the treatment efficacy of chemotherapeutics (5-fluorouracil, irinotecan, and bevacizumab) have also been evaluated.

### Carrageenan-based hydrogels

Derived from seaweed, carrageenan is composed of the basic units of the d-galactose and 3,6-anhydrogalactose. Carrageenan is classified into three types based on the number and position of the sulfate groups (λ, κ, and ι). Among these, κ-CG is the most common, accounting for more than 60% of all types.

Carrageenan may be utilized to prepare ionotropic and thermotropic gels. Due to the random coil-helix transition of chains, carrageenan exhibits a thermo-reversible solution-to-gel transition below a critical temperature. Upon cooling from high temperatures, ι-carrageenan undergoes only random coiling, rearrangement, and the formation of gel networks, whereas κ-carrageenan aggregates into helical structures after the formation of double helixes and the initial gels. Thus, the former is more likely to form soft elastic gels, whereas the latter is more likely to form mechanically stiff gels.

Because of their excellent gel-forming and high water-holding properties, carrageenan-based hydrogels, particularly κ-carrageenan hydrogels, have been prepared and applied for bone tissue regeneration or wound healing [174]. For example, κ-carrageenan was used to create a nanoengineered hydrogel system capable of electrostatically entrapping nanosilicates. The κ-carrageenan used in this study improved mechanical stiffness and biocompatibility [175]. The entrapped vascular endothelial growth factor can be continually released from hydrogels to accelerate hemostasis, facilitate tissue regeneration, and promote wound healing. This nanocomposite has tremendous potential for use in wound healing.

In another study, Jaiswal et al. developed κ-carrageenan-based hydrogels by incorporating chitosan capped sulfur NPs (SNP, promoting skin wound healing), and a powerful antioxidant compound (grapefruit seed extract) to exert synergistic effects of wound protection against microbial invasion and wound healing [176]. Within 3 h of incubation, the hydrogel coating exhibited potent antibacterial properties that completely eradicated *Staphylococcus epidermis* and *Escherichia coli*. Notably, the as-prepared hydrogel significantly stimulated the adhesion and proliferation of firming keratinocytes and reduced the wound area (from 31% to 1.3% of the wound area) in SD rats after two weeks of treatment, providing a promising strategy for wound healing.

In addition, carrageenan-based nanosystems could also be employed to improve the drug loading capacity, biodegradability, and achieve controlled drug release. [177]. In pursuit of this goal, a metformin hydrochloride (MET)-loaded hydrogel containing agar and κ-carrageenan was developed [178]. Positively charged MET attracted more readily to κ-carrageenan molecules with high sulfate content than agar, resulting in an increase in MET loading capacity. Notably, the agar hydrogel (without the κ-carrageenan component) demonstrated burst release of payload after 0.5 h and sustained release of up to 90% drug after 3 h, whereas the agar/κ-carrageenan mixed hydrogel demonstrated decreased burst release after 0.5 h and sustained release of MET after 9 h, indicating a prolonged drug release profile.

Despite the superb bioactivities of carrageenan, this kind of polysaccharide lacks specialized cell- or tissue-binding sites, which limits its application in nanotechnology engineering. Blending with other polymers with targeting moieties seems to be an essential point deemed worthy of consideration during the development of carrageenan-based nanosystems.

### Ulvan-based hydrogels

Ulvan, a marine sulfated heteropolysaccharide found in green algae, is composed of rhamnose, xylose, sulfate, d-glucuronic acid, and l-iduronic acid. Ulvan is utilized widely in biomedical, pharmaceutical, and cosmetic applications because of its great biocompatibility and broad bioactivities, which include antibacterial, immunostimulating, antioxidant, and anti-inflammatory effects [179, 180]. Hydrophilic ulvan can produce hydrogel in the presence of ions (such as B^+^ and Ca^2+^) at a basic pH, and their sulfate and carboxylate groups have a high water-absorption capacity [179]. Notably, the repeated aldobiouronic units of ulvan enhance the temporary “junction zones” formation, which is associated with weak gel formation. The ulvan-based hydrogels are biocompatible, hydrophilic, easily modifiable, water-absorption controllable, and porous in structure.

Ren et al. fabricated a natural polysaccharide-based hydrogel matrix comprised of ulvan dialdehyde, chitosan, dopamine, and anti-bacterial silver nanoparticles (Ag NPs), followed by the encapsulation human umbilical cord mesenchymal stem cells lyophilized powder (hUC-MSCs, used for promoting cell proliferation), yielding UC-DPA-Ag@hUC-MSCs hydrogel for accelerating diabetic wound healing [181]. The Schiff base reaction between aldehydes on ulvan dialdehyde and primary amines on chitosan formed the hydrogel matrix’s crosslinked networks. Besides, ulvan is a stabilizer for the preparation of silver nanoparticles that participates in the silver ion reduction process; therefore, the addition of ulvan to synthesized Ag NPs resulted in a highly stable colloidal system and prevented particle aggregation sterically. The therapy potentials of the hydrogel in a mouse model of type II diabetes with a full-thickness skin wound were indicated on Day 10 by a nearly completely healed wound, enhanced regeneration of cutaneous appendages, and an intact epidermal layer in the affected area. This as-prepared hydrogel provided a facile and effective strategy for diabetic wound treatment, hence facilitating the application of precious Ulvan materials in global and large-scale biomedical products.

### Agarose-based hydrogels

Agarose is a water-soluble linear polysaccharide derived from seaweed that comprises repeated agarobiose units (a disaccharide of D-galactose and 3,6-anhydro-l-galactopyranose). Its attributes could be summarized as follows: (1) It is believed that the presence of oxygen and hydrogen in agarose structures enables self-gelling behavior, which is favorable to the formation of functional hydrogel systems. (2) The porosity structure of agarose-based gels can be easily modified by altering the agarose concentration and additive selection during the crosslinking process in order to control the eventual loading and release behavior of payloads [28]. (3) The neutral surface charge of agarose suppressed the formation of protein corona on agarose-based nanomaterials, resulting in a reduction in nonspecific protein adsorption during blood circulation and an improvement in drug delivery efficiency. (4) Agarose gels provide mechanical support for cell adhesion and appropriate penetration of oxygen, water, and nutrients for cell growth and differentiation due to their structural similarity to extracellular matrix.

Agarose undergoes a reversible transition from solution to gel as a result of hydrogen bonding or electrostatic interaction-mediated cross-linking with heat-cooling progression. This progress in hydrogel production necessitates no additional toxic crosslinker, rendering agarose-based hydrogels biocompatible platforms [182]. Zhu et al. developed an injectable nanozyme hydrogel system that simultaneously encapsulating Prussian blue (PB) NPs and an aggregation-induced emission luminogen (AIEgen, CQu) in agarose hydrogels (Fig. 7C) [183]. Under irradiation with an 808 nm laser, PB was able to convert light into heat, hence leading to localized hyperthermia and reversible hydrogel hydrolysis and the subsequent CQu release. Following by the low-power light exposure, CQu generated high levels of ROS with sufficient O_2_, facilitating the tumor cell death. Due to the persisted retention of hydrogel at tumor site (for 48 h), the systems facilitated multiple rounds of treatment following a single injection, providing the clinical development of AIEgens treatment strategies for cancer patients.

In another study, a physically crosslinked supramolecular agarose/gelatin/polypyrrole/Fe^3+^ (AGP3) hydrogel was prepared by gelation through non-covalent interactions for spinal cord injury repair by filling the cavity and imitating the physiological properties of the spinal cord (Fig. 7D–i) [184]. Fe^3+^ was able to improve crosslinking densities, whereas agarose enhanced the thermal stability and mechanical properties of hydrogels (with good thermal-reversible properties), preventing the dissolution and enzymatic degradation of gels in vivo and maintaining matrix stability, which are conducive to regulating the morphology and regeneration of nerves. After spinal cord injury, the AGP3-treated group exhibited the greatest amount of freshly generated and mature neurons, as well as an increase in neural stem cell differentiation (Fig. 7Dii–iii). The combination of the three components endows the hydrogel with modulus and conductivity similar to those of the spinal cord, making it a desirable biomaterial for spinal cord injury repair.

Besides, introducing functional groups to agarose, like carboxymethyl modification, endowed the gel systems with improved surface properties, and promoted cell proliferation. For example, one study designed an innovative microporous hydrogel formed by carboxymethyl agarose and Ag^+^ via hydrogen bonding and supramolecular complexation dressing with anti-bacterial and anti-inflammatory properties [185]. The formulation could remarkably facilitate would healing attributing to the inherent interconnected porous structure allows blood and tissue exudates to be rapidly absorbed into the hydrogels. Due to the change of the state of agarose under temperature changes, Ag^+^ was gradually released as the temperature of the wound site increases, exerting antibacterial effect. The as-prepared hydrogel significantly accelerated skin tissue regeneration and wound closure in a cutaneous wound model.

Overall, agarose hydrogel provides an excellent matrix for different models of drug encapsulation. For a more controlled drug release profile, agarose can also be tailored with ROS-responsive, pH-sensitive, and thermo-responsive properties. The combination of agarose-based nanocomplexes with functional inorganic or organic constructions and high drug adsorption capability may provide future options for improving drug loading capacity and preventing off-target drug release.

## Marine structural protein-based nanocarriers

### Collagen-based nanocomposites

The majority of collagen used in commercial manufacturing is extracted from animal tissues (mostly pig or bovine tissue), while a small amount is also purified by recombinant synthesis systems [186, 187]. However, there is a critical concern about the potential risk of zoonosis (e.g., prions-induced mad cow disease, infectious spongiform encephalopathy, etc.) associated with collagen derived from mammalian sources. Over the past two decades, marine organisms have attracted considerable interest as a safe and reliable source of collagen extraction. Compared to bovine collagens, fish-derived collagens have higher viscosities, and fish collagen is characterized by the presence of two α-chains, namely α−1 and α−2 [188]. Fish collagen has lower levels of hydroxyproline and proline than mammalian collagen. It not only resolves the dilemma faced by the use of mammalian collagen but also fully improves the rate of reutilization of marine products and reduces environmental pollution.

Collagen, which is characterized by easy biodegradation, high biocompatibility, good bioactivity, and ease of processing, has been utilized in a wide range of applications, including but not limited to sponges or nanofibers for wound healing, microspheres, hydrogels, mini-pellets and tablets, and nanoparticles for drug delivery and tissue engineering [189]. Marine organism-derived collagen components could simulate the most abundant structural protein in the ECM of tissues, where it provides strength and structural stability for tissues, and then attract fibroblasts and keratinocytes to the wound site, thereby promoting angiogenesis and reepitheliogenesis during tissue injury [190]. Particularly, type II and IV collagen from cuttlefish and sponge possess ROS-scavenging ability, photoprotective activity, wound-healing capabilities, and alleviate degenerative osteoarthritis, hence promoting their synergistic therapeutic effect in tissue engineering [191]. As a proof of concept, Jridi et al. conducted a study in which they chemically extracted collagen components from cuttlefish skin byproducts in order to prepare collagen gels. The collagen gel exhibited a dense fibrillar microstructure, a highly interconnected network, and smaller pore sizes, as well as exceptional temperature reversibility, allowing it to adhere well to the skin’s surface [192]. When applied topically to mice with excisional wounds, the collagen hydrogel ointment effectively increased the wound healing rate on the 6 day of monitoring, and the percentage of wound contraction was 35.7% and 26.0% greater than the control and commercial drug groups, respectively. In addition, collagen gel application reduced infection and accelerated epithelialization. Furthermore, it has been demonstrated that the abundant methionine residues within the peptide chain of marine collagen hydrolysate serve as active sites for antioxidant activity [193]. Besides, it could scavenge ROS by inhibiting the ferroptosis signal pathway and depleting antioxidant GSH molecules, thereby enhancing cell viability when applied to tissue injury and aiding in the resistance to oxidation-induced cell death. In another study, it was shown that marine collagen hydrolysates obtained from the marine sponge C. reniformis and digested with trypsin had a significant ability to scavenge ROS by up to 60% [194]. These hydrolysates showed a favorable effect on keratinocytes and fibroblasts in terms of facilitating cell migration, indicating potential benefits in promoting cell migration or proliferation at the lesion site of epidermal and dermal cells and alleviating symptoms of various skin injuries. There is currently no marine sponge-derived collagen gel formulation for tissue regeneration, which should motivate researchers to further explore the design and application of related biomaterials. Besides, Howaili et al. prepared a low-cost wound dressing by incorporating Ag NPs into the decellularized fish swim bladder (mainly composed of collagen extracted from *Rutilus Frisii*) [195]. The dressing has wide-ranging antimicrobial properties and promotes cell growth. The porous structure of an as-prepared antimicrobial wound dressing is conducive to the growth of cells or the addition of other antimicrobial nano-sized materials for exerting wound healing effects in a synergistic manner.

Collagen can also serve as a natural substrate or scaffold for supporting new tissue. Langasco et al. proposed a novel biological dressing for skin wound healing in the form of powder or polymer membranes, which were prepared by loading L-cysteine hydrochloride, a sulfur amino acid well-known for its wound-healing properties, onto the collagen matrix of natural sponges [196]. In another study, Carvalho et al. conducted the synthesis of a hydrogel by blending solutions containing 5% collagen derived from jellyfish and/or 3% collagen sourced from blue shark skin, together with chitosan and 10% fucoidan [197]. The extracted collagen components possess a denaturation temperature ranging from 30 to 32 °C, together with a molecular weight (Mw) falling within the range of 120 to 125 kDa. Jellyfish and shark-derived collagen exhibits elevated levels of hydroxyproline and total proline, indicating their involvement in the formation of tissue triple-helical polypeptide connecting regions. These regions are stabilized by inter-molecular hydrogen bonds formed between carbonyl groups. Consequently, when incorporated into gels, these collagen types exhibit higher denaturation temperatures and increase mechanical properties and gel strength. Collagen provided appropriate signals that altered cell adhesion, viability, proliferation, and migration. Besides, the incorporation of both Jellyfish and shark-derived collagen into the hydrogel exhibited improved biomechanical stability. Specifically, the hydrogel with this combined composition experienced a mass loss of only 18% over a 30 day period. In contrast, hydrogels solely composed of jellyfish or shark-derived collage demonstrated higher mass losses, approximately 35% and 44% respectively. The potential application of hydrogel-encapsulated chondrocyte cells in cartilage tissue engineering is indicated by their vitality.

The high orientation of marine collagen is advantageous for guiding cartilage regeneration. These collagens can be utilized as substrates for cell culture, thereby promoting cell adhesion, cell proliferation, and chondrocyte differentiation. Therefore, the application of porous scaffolds comprised of marine collagen in bone tissue engineering has potential benefits.

Marine-derived collagen materials have been proposed as cell templates for tissue regeneration or wound dressing matrices. Nevertheless, there are still concerns regarding the host's immune response to these marine organism-derived components, and there is a lack of information regarding their immunogenicity in the literature. Recent studies have proven that collagen derived from blue sharks and codfish induced negligible expression of pro-inflammatory cytokines after *i.p.* administration in C57BL/6 mice, further providing evidence of the biosafety of collagen components from marine organism. However, additional evidence of the biosafety of marine-derived collagen is necessary before this promising biomaterial candidate can be utilized in regenerative medicine [198].

### Gelatin nanocomposites

Gelatin is extracted and purified from collagen components. As a result of the aromatic groups in collagen’s tertiary structures, collagen components readily induce undesirable immunological responses, and the collagen-based gels lack mechanical and thermal stability without additional crosslinkers. In comparison to their parent collagen compounds, gelatin-based hydrogels are superior in terms of self-healing, biocompatibility, biodegradability, and reduced cytotoxicity [157, 199]. Compared to gelatin derived from mammalian, fish gelatin contains less proline and hydroxyproline, resulting in lower gelation and melting temperatures. Therefore, fish gelatin-hydrogels might be fabricated at relatively low preparation temperatures (such as room temperature), making them more appropriate for loading with heat-sensitive therapeutic drugs or fabricating processes requiring low temperatures. Due to their high viscosity and low mechanical properties, fish gelatin hydrogels are more favorable for constructing specific tissue analogs in the engineering of soft tissue applications [200, 201].

Chemical (generally mediated by glutaraldehyde) or enzymatic (e.g., transglutaminase and tyrosinase) crosslinking is required for the covalent crosslinking of gelatin. However, enzymatic crosslinking makes it difficult to modulate the crosslinking density, which hinders the modification of its mechanical properties. Functionalization of gelatin (such as modification with methacrylate groups) prior to crosslinking could modulate the crosslinking density of gels, hence offering an alternate strategy [202, 203]. By utilizing elevated methacrylate gelatin concentrations, it is possible to obtain enhanced mechanical strength, however, this advantage comes at the expense of compromising the material's porosity, degradability, and its ability to facilitate 3D cell attachment. Besides, methacrylate gelatin could be incorporated with natural biopolymers (like silk fibroin, HA, etc.) or synthetic polymers (polyacrylamide, polyaniline, polycaprolactone, and so forth) to reinforce the mechanical properties tunning from tens of kPa to hundreds of kPa.

**Fig. 8:**
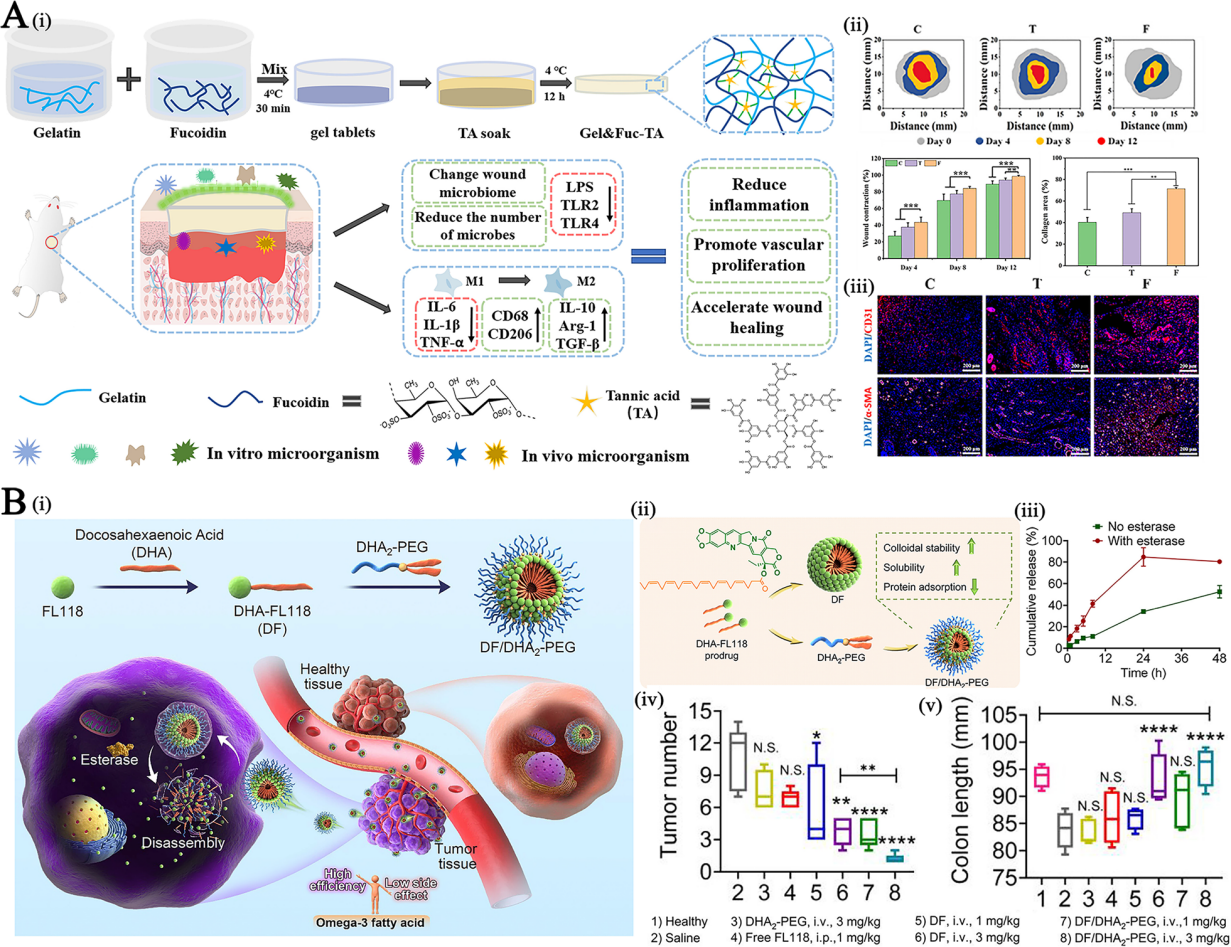
**A** (i) Schematic of the tilapia fish skin gelatin-fucose gum-tannic acid (Gel&Fuc-TA) hydrogel and treatment mechanism for wound healing (ii) The wound healing and quantified wounds contraction ratio in rat skin incision model after different treatments; (iii) The fluorescence expression of platelet endothelial CD31 (vascular endothelium-specific marker) and α-SMA (a vascular smooth muscle cell marker). Reproduced with permission. [205]. Copyright 2022 Elsevier B.V. **B** (i) and (ii) The formation of omega-3 fatty acid-derived nanocarrier self-assembled by amphiphilic PEG-docosahexaenoic acid (DHA2-PEG) conjugate and DHA-camptothecin (e.g., FL118) prodrug; (iii) The tumor numbers in colon and (iv) colon lengths in murine model of colitis-associated carcinogenesis. Reproduced with permission. [214]. Copyright 2022 Elsevier Ltd

Chen et al., prepared multifunctional fish-derived scaffold composed of methacrylate fish gelatin, decellularized fish scale, while black phosphorus nanosheets and mesenchymal stem cells were incorporated into the network [204]. The as-prepared scaffold promoted regeneration of mice calvarial defect in vivo, suggesting a promising strategy in bone regeneration. However, chemical crosslinking usually requires additional chemical reactions, and the introduction of organic solvents is unavoidable. To solve the problem, Lu et al. used a green and simple method for the gelatin hydrogel preparation without the introduction of chemical groups or external stimulation (Fig. 8A–i) [205]. Tilapia fish skin gelatin and fucoidan were immersed in a tannic acid (TA) solution at a low temperature (4 °C) to form hydrogel wound dressings (Gel&Fuc-TA) via physical cross-linking between TA and gelatin, fucoidan. The Gel&Fuc-TA hydrogel exhibited higher swelling properties at pH 7.4 and achieved a continuous release in 196 h at pH 7.4, as the deprotonation of TA in a weakly alkaline environment caused hydrogen bond fracture, leading to reduced cross-linking strength between hydrogels. The intrinsic biomedical activities of fish skin gelatin and the other two components, along with the hydrogel’s complex porous structure, assured good complementary effects in biocompatibility, hemostasis, and inflammation management. The topical administration significantly promoted wound healing (with 92% contraction of the wound area), increased collagen deposition in the rat skin incision model, and prevented massive bleeding and promoted blood vessel formation in the rat tail break model and rat hemorrhagic liver models (Fig. 8Aii–iii). This formation does not require further chemical reactions to produce biomedical materials, broadening the investigation of biological resource by-products for biomedical applications.

The application of marine organism-derived gelatin can reduce the cytotoxicity, immunogenicity, and side effects of gelatin-based nanosystems, making them eco-friendly and decreasing the production cost. However, commercial marine-derived gelatin products are mostly used in the food industry, cosmetics, or as dietary supplements. There are still no commercial products of marine-derived gelatin focused on drug delivery systems for disease treatment. Recent investigation of the topic of gelation has rapidly shifted the focus on developing multicomponent gelatin nanocomposite hydrogel for building better tissue substitutes and stimuli-responsive smart hydrogels to further expand the application of gelatin derived from marine organisms.

## Marine food sources-derived fatty acid-based nanomaterials

Fish oils are enriching in omega-3 fatty acids (i.e., docosahexaenoic acid (DHA), eicosapentaenoic acid (EPA), and α-linolenic acid (ALA)), which are well known bioactive dietary compounds [26]. Adequate intake of those polyunsaturated fatty acids (PUFAs) may provide various health benefits, including anti-inflammatory, antioxidant, anti-cardiovascular abnormalities, antithrombotic, antidiabetic, and hypolipidemic effects [206, 207].

### Marine-derived fatty acid NPs for enhancing anti-tumor effects

It has been reported that the inhibition of tumors by EPA and DHA is dose-dependent and achieved through multiple mechanisms. Inducing cell cycle arrest and apoptosis, inhibiting the arachidonic acid pathway, and inhibiting angiogenesis are among these mechanisms. It has been reported that omega-3 PUFAs mechanically stimulate the PI3K/Akt signaling pathway, resulting in the up-regulation of the tumor-suppressing protein OKL38 [208]. This upregulation leads to increases in the Bax/Bcl-2 ratio and the release of cytochrome C from the mitochondria, resulting in cancer apoptosis. By inhibiting cytokines such as vascular endothelial growth factor (VEGF) and platelet-derived growth factor (PDGF), n-3 PUFAs have the potential to decrease tumor angiogenesis. Additionally, it can hinder tumor invasion and metastasis by reducing tumor cell rolling, adhesion, and migration. Importantly, previous research has shown that DHA can increase the susceptibility of cancer cells to chemotherapeutic agents without amplifying the toxicity to healthy tissues [209, 210]. Both of these factors have prompted researchers to incorporate marine-derived PUFAs into drug delivery systems as assemblers or drug loaders for the purpose of synthetically enhancing cancer treatment. Notably, based on the role of tumor cells in the uptake of natural fatty acids as biochemical precursors and energy sources, the combination of EPA and DHA with nanomaterials has the potential to enhance tumor cell uptake of carriers. This, in turn, can contribute to higher concentrations of cytotoxic drugs in tumors and prolonged drug activity.

Some evidence has demonstrated the beneficial effects of dietary DHA toward anti-cancer treatment due to its potent, dose-dependent cytotoxic effects on tumor cells. More encouragingly, in phase I clinical trials, DHA-Paclitaxel conjugate (Taxoprexin) demonstrated excellent anti-tumor activity and a favorable cytotoxicity profile toward tumor cells [211, 212]. Given the anti-tumor effects of this long-chain lipophilic fatty acid, DHA, Wen et al. designed low-density lipoprotein (LDL) NPs with a small size (20 nm) and negative surface charge (surface charge: − 25 mV) that were uniformly reconstituted with unesterified DHA (namely LDL-DHA) [213]. After local and regional transarterial administration into the liver, LDL-DHA could selectively kill hepatoma cells and reduce the growth of orthotopic tumors without histologic or biochemical injury to normal tissues, resulting in greater than 80% necrotic tumor tissue in hepatocellular carcinoma-bearing rats. After LDL-ADH is taken up and degraded in tumor cells, the bisallylic hydrogens of DHA are attacked by ROS, causing massive lipid peroxidation and cell membrane damage, providing an attractive therapeutic alternative for locoregional cancer indications.

Jiang et al. prepared injectable nanosystems (DF/DHA_2_-PEG, size: ~ 145 nm, zeta potential: − 10 mV) assembled by amphiphilic DHA_2_-PEG and DHA-monoconjugated FL118 (a camptothecin analog) prodrug (via an ester bond) (Fig. 8B–i and Fig. 8B–ii) [214]. Tethering the DHA motif to the drug promoted controlled release and the strength of the interactions between the molecules (potential intermolecular π-π stacking and hydrophobic interactions between polyunsaturated alkyl chains of DHA), resulting in NPs with a low critical micelle concentration and sufficient in vivo stability. The oral administration of DF/DHA_2_-PEG nanoassemblies inhibited colonic carcinogenesis and burden (as indicated by a significant reduction in tumor volume and number) in colitis-associated mouse models of colorectal cancer (Fig. 8Biii–iv).

### Marine-derived fatty acid NPs for facilitating transport across the blood brain barrier

Additionally, DHA and EPA have been extensively studied for their beneficial effects on neurological development and function, and these components are generally supplied to the brain and eyes predominantly from dietary sources [215, 216]. They could prevent neuronal damage that is related to aging or neurodegenerative diseases [217]. The brain lacks the ability to endogenously synthesize DHA and hence relies on exogenous sources, such as the circulation, to acquire this essential nutrient. This acquisition occurs through the process of traversing the blood–brain barrier (BBB). The DHA nutrient has been observed to be efficiently transported across the BBB through the action of the major facilitator superfamily domain containing 2A (Mfsd2a), which is a membrane transporter mostly located in the endothelial cells of BBB microvessels. This transportation process is dependent on the presence of sodium ions [215, 218]. This highlights the potential of incorporating DHA into nanocarriers to improve the penetration of pharmaceuticals across the BBB and the accumulation of formulations in the brain via the active transport mechanism facilitated by the Mfsd2a membrane transporter.

One study has reported the potential benefits of incorporating DHA (containing 5–15%) in lipid nanocarriers (90–140 nm in size) for increasing the BBB passage of the payloads (darunavir, antiretroviral drugs) [219]. With the increasing addition of DHA, the NPs achieved increased uptake, while NPs with higher DHA content (15%) reached up to ninefold higher permeation than free drug in vitro. Besides, the addition of DHA further facilitated the brain drug accumulation over the free drug with about a sixfold increase, supporting the development of DHA-based nanocarriers as an effective, safe, yet technically simple strategy to enhance brain delivery of drugs.

### Marine-derived fatty acid NPs for inflammatory disease treatment

Ingestion of EPA and DHA could significantly reduce the release of arachidonic acid (AA, which is involved in the pro-inflammatory response) from cell membrane phospholipids involved in inflammation, thereby inhibiting the inflammatory metabolic signaling pathways [220]. Specifically, EPA and DHA decrease the production of pro-inflammatory mediators such as prostaglandins-2, leukotrienes, hydroxyl eicosatetraenoic acid, and epoxy eicosatrienoic acid, which are produced by enzymes such as cyclooxygenase-2 (COX-2), lipoxygenase, or cytochrome P450, thereby inhibiting the inflammatory cascade. Besides, EPA and DHA have been proven to increase resolvins, and DHA has been shown to enhance protectins and maresins, which both have potent anti-inflammatory properties and promote the resolution of inflammation. As a special lipid component, PUFAs such as EPA or DHA can be directly incorporated into lipid nanocarriers, which allows for a prolonged circulation time in vivo and effectively prevents oxidative degradation. Compared to fish oil as a dietary supplement, which typically requires a higher dose, formulations based on PUFAs would enhance the efficacy of drug delivery to lesions. This improvement would reduce the administration dose and improve bioavailability, thereby maximizing the synergistic anti-inflammatory effects.

Considering the anti-inflammatory benefits of omega-3 fatty acids, a liposome incorporating the superparamagnetic nanoparticle Nanotex, fluorescent dye (rhodamine-100), and omega-3 PUFA was developed for imaging-guided drug delivery [221]. This formulation combined the treatment effects of omega-3 PUFA with the non-invasive multimodal imaging functions of superparamagnetic NPs and fluorescent dye. The liposomes proposed here have been shown to have significant treatment effects, regarding ameliorating inflammation and slowing down the proliferation of tumor cells, respectively.

Marine-derived lipids, especially the aforementioned omega-3 fatty acid components, provided physiological significance and potential health benefits. Recent research has focused on the encapsulation of omega-3 PUFAs in different colloidal particles to improve their dispersibility, stability, and bioavailability. However, the application of these PUFA as assemblies for nanoparticle formulation is still rare. As lipophilic fatty acid chains, those PUFAs could be used as hydrophobic portions of block copolymers for nanocarriers or as matrix components of emulsions and nanoemulsions, providing significant flexibility in nanoformulation design.

## Marine biomineralized nanocomposites for biomedical application

### Calcium carbonate-based nanocomposites

Calcium carbonate (CaCO_3_), the main component of mollusk shells or coral skeletons, possesses biodegradability, excellent mechanical strength, and a porous nature that makes it suitable for biomedical applications such as bone regeneration.

The calcium carbonate mineral phase in the coral structure exhibited tubular and slit-like pores, which are similar to those in natural bones and suitable for cell colonization and invasion of blood vessels. Recent studies have demonstrated their beneficial roles in osteointegration and osteoinduction, as well as their ability to induce functionally vascularized bone grafts. The incorporation of CaCO_3_-enriched inorganic (coral) materials into bone tissue-engineering scaffolds could significantly promote osteoblast-scaffold interactions, resulting in enhanced cell viability and more well-defined cell morphology [222].

In addition, due to its high surface area, large surface structural porosity, large loading capacity, pH-dependent drug release, biocompatibility, and biodegradability, the CaCO3-based vehicle is also an ideal drug delivery system. For example, aragonite CaCO_3_ derived from cockleshell was used as a matrix of pH-responsive carriers for controlled drug release [223, 224]. The loaded drugs were sustained release in physiological conditions for several days, but the payloads were rapidly released in an acidic tumor microenvironment due to the pH sensitivity of CaCO_3_ nanocrystals. Moreover, CaCO_3_ NPs decomposed into Ca^2+^ and CO_2_ in an acidic environment, with the released Ca^2+^ having the potential to further facilitate calcium overload-induced tumor cell apoptosis. This also indicates that the vehicles themselves may have antitumor synergistic potential during cancer therapy.

### Calcium phosphate and hydroxyapatite-based nanocomposites

Calcium-phosphate (CaP), an inorganic mineral of hard tissues, dominates the structure of the skeleton and dentition of many marine vertebrates. CaP is considered an excellent alternative in biomedical and implant applications due to its similarity of physiochemical properties to those of natural bones and teeth. Besides, the tailorable biodegradability, mechanical stability of CaP materials allows them to be an excellent option in orthopedics and dentistry applications. Additionally, the CaP nanoparticles were also fabricated and applied in the delivery of therapeutics, attributing this to their controllable particle size, enhanced surface-to-volume ratio, mild preparation conditions, and pH responsiveness, promoting their application in drug delivery [225–227].

Hydroxyapatite (HAp) is a colloidal form of calcium phosphate, essentially a highly polymerized polymer of calcium phosphate. These HAp could be fabricated on a nanoscale with an enhanced surface-to-volume ratio and more advantageous mechanical properties and exclusive biological functions [228]. Unlike the synthetic nano-HAp that induced toxicity toward normal tissues or cells, the biogenic HAP extracted from marine sources that possess mineral ions and porous architecture mediated better biocompatibility and bioactivities (such as bone bonding abilities) in vivo [229]. For instance, a 20 nm nano-HAp was synthesized from the bones of discarded Sardinella longiceps, and the as-synthesized particles demonstrated excellent biocompatibility. The nano-sized HAp provided a greater surface area per HAp volume for osteoblast adherence and proliferation and thereby played a role in increasing bone mineral density [230].

HAp could also be prepared as porous microspheres with high drug loading. Using the hydrothermal method, Huang et al. successfully created a bio-HAP porous microsphere with uniform morphology from an abalone shell [227]. As a result of the increased degradation rate of HAp with the gradually decreasing pH, the cumulative release of the payloads (DOX) exhibited increased trends under an acidic pH value. The porous microspheres prepared here possess a high drug loading efficiency (~ 95.5%) and a controlled drug release profile, and also induced a high level of apoptosis in tumor cells, which provides potential application value in tumor therapy.

All these proposed platforms (CaCO_3_, CaP, and HAp nanocomposites) are eco-friendly and low cost as the raw materials are directly extracted from marine mollusk shells, fish bones, or coral skeletons, which gives a second chance for the use of the large amounts of waste or by-products from the fishing and food industries.

Currently, aragonite, calcite, and vaterite are the most prevalent forms of CaCO_3_ [231, 232]. The most common chemical synthesis method employs co-precipitation, or double decomposition, and carbonation of CO_2_ gas through calcium hydroxide to produce a mixture of calcite and vaterite with poor biocompatibility. In contrast, shellfish from natural marine sources, such as cockles, are a valuable source of CaCO_3_ in the form of biogenic aragonite polymorphs, resulting in CaCO_3_ structures with higher purity and better biological compatibility [233]. Although the aragonite-type structure derived from marine shellfish is slightly less thermodynamically stable, it is more sensitive to pH changes, making it more appropriate for the preparation of stimuli-responsive nanomaterials.

Besides, the extraction of HAp through a simple calcination process does not require the use of toxic chemical solvents or the production of byproducts from chemical reactions, making it safer than the production of HAp through a chemical reaction between calcium and phosphorus-containing compounds. These points promote the development of marine biomineral-based nanodrug delivery vehicles for biomedical applications.

## Conclusions and outlooks

The evolution of high-value biomaterials and their synthetic derivatives derived from marine resources has accelerated over the past decade. Due to the unique marine environment, which includes high salinity and limited dissolved oxygen in the seawater buffer system, as well as low temperature, less light, and a minimal temperature difference, marine resources (polysaccharides, proteins, lipid components, etc.) evidently differ in structure and composition from those derived from terrestrial species. In contrast to terrestrial polysaccharides, a number of marine polysaccharides are highly sulfated, especially in the cell walls of macroalgae. Extensive scientific research has consistently demonstrated the superior biological properties (including functions related to immunity enhancement, antiviral effects, and antioxidation) of sulfated polysaccharides in comparison to their non-sulfated counterparts [234, 235]. As another example, marine collagen or gelatin has lower gelling and melting temperatures than those from traditional terrestrial sources of collagen or gelatin. Besides, collagen derived from marine fish consists predominantly of type I collagen, which provides a rich source of lysine and proline. This collagen component extracted from marine sources possesses a reduced molecular weight, facilitating enhanced absorption and consequently leading to increased bioavailability. Hence, the distinctive biological properties exhibited by marine-derived materials render them highly valuable biomaterials in the field of biomedical applications.

There are still important aspects of nano/microstytem prepared from marine-derived components that need to be investigated in future studies, such as:

1. First of all, it is necessary to consider that the raw materials of these formulations possess different microstructures and physicochemical properties due to their various sources, making it difficult to make a standardized distinction. Besides, the varying extraction and purification processes of raw materials from marine organisms lead to batch-to-batch variations and would affect the in vivo pharmacokinetics and pharmacodynamics results of the prepared biomaterials. These issues make it difficult to meet the stringent quality control requirements of prepared biomaterials in medical applications.
2. For chemical modification and modification aspects, some functional groups or copolymers are usually incorporated or grafted into the main chain structure of the raw materials to further improve their therapeutic effects. However, it is unknown whether these modifications will affect the original therapeutic properties of the materials or cause other side reactions.
3. The single performance increase of those particles (such as physiological stability/biodegradability, drug encapsulation/loading efficiency, in vivo drug biodistribution, etc.) might no longer meet the requirements of clinical application. For further controlling drug delivery, several unique chemical functionalities were further embedded in those aforementioned marine biomaterials, e.g., ion-binding groups, stimuli-responsive capabilities, traceability, or active targeting abilities, yielding multifunctional nanocomposites. However, those properties are still not satisfactory when facing complex physiological environments. The properties and functions of several marine materials should be further explored and applied, such as the P-selectin targeting of fucoidan and CD44 receptor-targeting of CS, and the pH-sensitivity of chitosan, alginate, or calcium carbonate nanocrystals. These materials could be combined with other stimuli-responsive conjugates or groups to form cascaded-responsive nanoplatforms with multiple response modes. Thus, those cascaded-responsive nanoplatforms could achieve point-to-point breakthroughs across different physiological barriers, resulting in an interlocking process of drug delivery.
4. Several researchers have attempted to design marine polysaccharide-based nanocomposites for the delivery of two types of drugs while also combining the polysaccharide's inherent biomedical properties to achieve combinational therapies. However, the question of how to combine those therapeutics rationally, quantitatively, compatibly, and synergistically into one assembled vehicle without affecting their original functions remains unanswered.
5. Particularly, the mechanism studies of those marine biomaterials for “precision” nanomedicine are waiting to be investigated in further works. That is, the tissue localization of these marine material-based nanoformulations and the intercellular localization of the payload should be carefully studied. Besides, it is still required to conduct more clear studies that mainly focus on the relationship of the physicochemical properties of vehicles (e.g., material composition, surface charge, particle size, particle morphology, aggregation state, etc.) with their in vitro and in vivo functions (e.g., drug release manners, biodistribution, therapeutic efficiency, the metabolic pathways, toxicity in vivo, etc.).
6. It is also important to apply a continuous, highly scalable, and reproducible fabrication process to those marine material-based nanoformulations. Green, simple preparation techniques, such as one-pot precipitation methods, FNC technology, and so on, provide a reference for achieving high-value particles with controlled particle size, uniform composition, high colloidal stability, tunable physicochemical properties, are highly scalable and reproducible, have prolonged storage stability, have good performance consistency, and so on, which are also important for their future applications.
7. The biosafety of marine biomaterial-based nanocomposite materials is still a major concern among the public due to the lack of clinical data on their safety and efficiency in human bodies. The biosafety evaluations of these marine-biomaterial nanocomposites are still concentrated on in vitro or preliminary animal studies. There are few studies, in particular, that fully emphasize those clinically important indices: oncogenicity, genetic toxicity, allergic effects, mutagenicity, reproductive and developmental toxicity, and organellar damage of those formulations. The marine organisms from which biomaterials are extracted, on the other hand, come from a variety of sources with unknown immunogenicity. All of the previously mentioned biosafety concerns necessitate additional systematic testing using various large animal models, as well as accelerating clinical testing of approved products. For those specific formulations (i.e., sustained-release drug delivery systems), the nanodrugs commonly have long half lives in human bodies, which are liable to concentrate toxic degradation products of materials in vivo. When those long-acting formulations are used in chronic disease treatment that requires repeated administration, those materials might accumulate in the tissues or organic via bioaccumulation and biomagnification. The toxic components could induce side effects and hypersensitivity reactions once a certain threshold is exceeded in the human body while also attenuating the positive effects of formulations. The preclinical trial designs shall be adjusted and harmonized for continuous assessment of the relationship between their efficacy and toxicity in vivo over a long period (for several months or even one year).
8. Specifically, even though there are few chitosan- and alginate-based materials applied in wound dressing and hemostasis in an emergency on the market, no relevant patents or products have been applied in clinical trials of nanoscale chitosan biomaterials used for drug delivery.

In conclusion, biomedical research on marine material-containing drug delivery systems has a promising future due to their abundance of raw materials, high biocompatibility, flexible structures, simple preparation processes, and low toxicity. Further research should focus on optimizing purification procedures for various marine-sourced raw materials. In addition, it is essential to establish detailed and standardized quality assurance and quality control methods and systems for raw materials. To maximize therapeutic benefits, it is also necessary to develop multifunctional drug delivery systems based on marine materials that can precisely target specific cells or organelles, enable cascade responses, and combine multiple disease therapeutic targets, allowing for flexible regulation of drug release patterns and synergistic therapeutic effects. In addition to cost and practicability, it is essential to consider the reproducibility of material preparation across different batches. Furthermore, the process of large-scale preclinical trials should be expedited and coordinated to continuously evaluate the long-term relationship between in vivo efficacy and toxicity, thereby facilitating clinical translation. Once those aforementioned issues are solved, those formulations would provide powerful approaches for better overcoming long-standing scientific and technical challenges in biomedical applications. It is hoped that this review could provide an overall framework for the research progress of functional marine biomaterials as well as attract more academic researchers and companies to invest more efforts in comprehensive and multifaceted research, eventually promoting the process of clinical transformation.

## Data Availability

Not applicable.
